# High variability of food and nutrient intake exists across the Mediterranean Dietary Pattern—A systematic review

**DOI:** 10.1002/fsn3.1784

**Published:** 2020-07-29

**Authors:** Asmaa Abdelhamid, Amy Jennings, Richard P. G. Hayhoe, Veronica E. Awuzudike, Ailsa A. Welch

**Affiliations:** ^1^ Norwich Medical School University of East Anglia Norwich UK

**Keywords:** dietary patterns, food groups, macronutrients, Mediterranean Diet, Mediterranean Diet score, micronutrients, review

## Abstract

A Mediterranean style dietary pattern (MDP) is considered beneficial for health. The MD Score (MDS) definition has evolved, resulting in considerable variability in the foods and nutrients associated with MDS adherence. We systematically investigated food and nutrient composition of the MD between studies, countries, and methods of classifying the MDS. We searched Embase for MD systematic reviews and selected observational studies reporting intakes of foods, macronutrients, or micronutrients by categories of MDS adherence. The percentage differences in food and nutrient intakes between categories of high and low adherence to the MDS were calculated for each study. A total of 369 full‐text primary papers were reviewed from the included systematic reviews and 74 papers selected (66 adults, 8 children). We found considerable differences in MDS definitions and scoring criteria. Between‐study variation in food intake between high‐ and low‐adherence MDS adherence categories ranged from a mean of −23% for meat, to 119% for fruit, and 278% for fish. Greater variability was evident in non‐Mediterranean than Mediterranean regions. We conclude that few studies report food and nutrient intakes across the range of the MDP in adults and even fewer in children. The considerable variability in the foods and nutrients reported makes comparison of results from studies and translation into dietary guidelines difficult. We recommend that future publications of MD studies include full details of the range of food and nutrient intakes across the distribution of MD adherence in order to facilitate translation into health policy and practice.

## Graphical abstract

While considered beneficial for health, the Mediterranean Diet definition has evolved, resulting in considerable variability in the foods and nutrients associated with adherence. We reviewed food and nutrient composition of the MD between studies, countries, and methods of classification and found considerable variability in the foods and nutrients reported which makes comparison of results difficult. Future MD studies should include details of a full range of food and nutrient intakes by categories of MD adherence in order to facilitate translation into health policy and dietary guidelines.

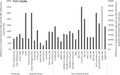

## 1 INTRODUCTION

The Mediterranean dietary pattern (MDP), widely used in nutritional epidemiology, has been suggested to protect against a number of noncommunicable diseases. However, while there is strong evidence that a Mediterranean Diet (MD) reduces risk of cardiovascular disease (Liyanage et al., 2016) and diabetes (Koloverou, Esposito, Giugliano, & Panagiotakos, 2014), evidence from studies investigating associations with other disease are weaker. This includes conflicting or inconclusive data regarding stroke risk (Rosato et al., 2017; Tong, Wareham, Khaw, Imamura, & Forouhi, 2016), fracture incidence (Craig et al., 2017), cognition (Anastasiou et al., 2017; Cherbuin & Anstey, 2012), blood pressure (Davis et al., 2017), and type II diabetes (Ceriello et al., 2014; Esposito et al., 2009).

The MDP is a pre‐defined dietary pattern that includes a high intake of fruits and vegetables, legumes, nuts and seeds, olive oil and wholegrain cereals with moderately high intakes of fish, and regular moderate intakes of alcohol, low to moderate intakes of dairy products and low intakes of saturated fats and red meats (Trichopoulou et al., 2014). The original Mediterranean Diet Score (MDS) was based on patterns of eating in Greece (Trichopoulou et al., 1995) but has subsequently evolved, with a further 28 variations now in use, resulting in considerable variability in the method and the categorization of food groups and nutrients included within the scores (Craig et al., 2017; Davis, Bryan, Hodgson, & Murphy, 2015; Hernandez‐Ruiz et al., 2015; Olmedo‐Requena et al., 2019; Shaw, 2016; Zaragoza‐Marti, Cabanero‐Martinez, Hurtado‐Sanchez, Laguna‐Perez, & Ferrer‐Cascales, 2018). It is also now used in non‐Mediterranean and Mediterranean regions.

The technical construction of the MDS takes into account the distribution of food categories within a population and classifies intake based on country or study‐specific percentile cut points. As the value of the cut points used to define the categories within the MDS depend on the distribution of the food and food group intake, within a population, the nutrient composition of the MDS will vary between studies. For example, fruit and vegetable intake varies from northern to southern Europe with lower total intake in the North compared to the South (Agudo et al., 2002; Slimani et al., 2002). Moreover, while the predominant vegetables eaten in the North are brassica and root vegetables, consumption of the fruiting vegetables such as aubergines and peppers predominates in the South with consequent impact on nutrient intakes found within the vegetable category when generated within different countries such as carotenoids (Agudo et al., 2002; Jenab et al., 2009; Slimani et al., 2002). These geographical differences also extend to other foods such as dairy products, meat, fish, and cereal foods (Slimani et al., 2002; Welch et al., 2002, 2009). Understanding the composition of the foods and nutrients within the MD is important as it is their synergistic activities of nutrients that are thought to confer the health benefits of this dietary pattern (Paterson Katherine et al., 2018). This is also important as the MDP forms the basis for health recommendations and policy with consequences for agricultural production. In addition, as the MD recommends lower intakes of meats and dairy foods, this could impact on musculoskeletal health through effects on nutrients such as protein and calcium considered important that are considered important (Craig et al., 2017) but are frequently scored negatively within the MDS. While the MDP is considered a consistent pattern of eating, there is potential for large variability in the food and nutrient composition of the MDS due to the methods of score construction as well as to the geographic variation in intakes of types of foods. The aim of our study was to understand the variability in food and nutrient intakes according to categories and across the distribution of MDS adherence and to understand whether this variability may differ across regions and methods of classifying the MDS.

## 2 METHODS

We systematically reviewed the literature to identify papers that have published data on categories of intake of the Mediterranean Diet (MD), describing the full range of the MD that provide details of the foods and nutrients of interest. The PRISMA (Preferred Reporting Items for Systematic Reviews and Meta‐Analyses) checklist (Moher, Liberati, Tetzlaff, & Altman, 2009) was used to guide reporting.

### 2.1 Search strategy

An initial scoping literature search was conducted to identify the published food and nutrient intakes according to MD adherence. The scoping search was conducted in PubMed and Google Scholar on the 24 January 2017, using search terms such as “Mediterranean Diet AND dietary intake” six papers complied with the inclusion criteria were found. Due to the anticipated vast number of publications on MD, we limited our search to systematic reviews of MD studies. A structured search strategy was then developed and focused on searching for all systematic reviews of relevant studies. The search was conducted in Embase on 16 April 2018. It was unrestricted by date and used the MeSH term “Mediterranean Diet” without focusing or exploding. The search was limited to studies in humans, English language, Embase, and systematic reviews.

### 2.2 Study selection

PICOS criteria for inclusion of studies in the review are detailed in (Table 1). The study selection was done in two‐stages. One author (AA) first screened and selected the potentially relevant systematic reviews. We then used our inclusion criteria to screen all papers included in the selected set of systematic reviews. We included studies of observational design (longitudinal cohort or cross‐sectional). Eligible studies had to have defined the MD a priori, have recorded the method of adherence to components/categories of the MDS, to have reported the data according to quantiles of adherence, as well as the observed mean or median dietary intake of at least one food group, macro or micronutrient according to these quantiles. We also included studies that reported quantile adherence data but with no variance of the mean or median of dietary intake. We excluded studies reporting only energy or alcohol intake (however, studies reporting any food group intake as %E intake were included). Studies, where intake is reported by a condition, an outcome or characteristic, for example, reporting MD adherence of obese versus normal weight or people with stroke versus not, were excluded as these were not considered representative of general population intakes. We also excluded data for studies that reported a range of consumption rather than the mean or median data for intake. Studies reporting on children or adolescent cohorts were selected and described separately.

**TABLE 1:** PICOS criteria for inclusion of studies in the systematic review

PICO component	Description
Population	Studies of people of any age, in any country, with any clinical condition where Mediterranean Dietary Pattern (MDP) was defined a priori
Intervention	No intervention (interventional studies were excluded)
Comparators	N/A
Outcomes	Reported observed mean or median dietary intake of any food group, macro‐, or micronutrient according to MDP adherence quantiles. Studies reporting only energy intake or alcohol intake were excluded
Study design	Observational design (longitudinal cohort or cross‐sectional)
Language	English

### 2.3 Data extraction

The following information was extracted from each study: age, sex and of participants, sample size, study design, cohort details including location and dates of dietary data collection and the proportions of M/F, and whether combined data were reported. Also extracted were the type of MD score/index used, the primary reference for the dietary score, intake data variables available (foods and nutrients), groups of adherence within the scores and the dietary assessment method. Dietary intake according to quantiles of MD adherence was extracted and separated into three major categories: food group, macronutrient, and micronutrient intakes. Only data from the highest and lowest quantiles of adherence were recorded to highlight the range in MD adherence within populations. Food intake data were extracted from the following food categories used in construction of the MD: olive oil, fruit, vegetables, legumes, red meat, poultry, dairy, nuts, fish, and cereals as these were the components found in the traditional MDS (Trichopoulou, Costacou, Bamia, & Trichopoulos, 2003). Intake values were recorded for these macronutrient categories: energy, fat, alcohol/ethanol, protein, carbohydrates, MUFA, PUFA, SFA, and fiber intake. Micronutrient intake included the following: vitamin B, where vitamin B intake was combined with folate thiamin and all vitamin B complex, vitamin C and vitamin D, sodium, potassium, magnesium, calcium, selenium, iodine, iron, and zinc. Analysis was performed in Microsoft Excel. For food categories within the MD that are scored positively, olive oil, fruit, vegetables, legumes, nuts, fish and cereals, it was expected that an increase in consumption would be found across increasing quantiles of adherence to the MD, in contrast to those food groups scored negatively, that is red meat, poultry, dairy which would decrease.

### 2.4 Data analysis

To avoid multiple counting of data from studies within the same cohorts, studies were prioritized according to the following hierarchy: papers reporting on the largest cohort numbers and papers reporting clearly on different subsets of the cohort (e.g., EPIC). Further papers from the same cohorts were included in the analysis only if they reported additional data. Data from the studies were reported for intakes of foods and nutrients in various ways (e.g., servings, grams, % E) with differences even within these methods of reporting (e.g., different serving sizes, serving size not reported, or reported as % energy while total energy intake was not reported). Studies also reported categories/quantiles of MD adherence differently ranging from tertiles to quintiles, and within these categories, they reported data for means, medians, or frequencies. To overcome these large differences in reporting, allow comparison across data from different studies, and maximize the use of available data, we calculated the proportional differences across extreme quantiles. These were the differences between the highest and lowest quantiles of adherence to the MD. These proportional differences were calculated as the percentage difference between the highest and lowest quantile of adherence to the MD. The spread, mean, maximum, and minimum values of these proportional differences were calculated across studies for the highest and the lowest quantiles in the following groups; all included food groups, macro‐, and micronutrients (after removing duplicate cohort data). We also compared data from studies in the Mediterranean region with data from non‐Mediterranean regions.

## 3 RESULTS

**FIGURE 1:**
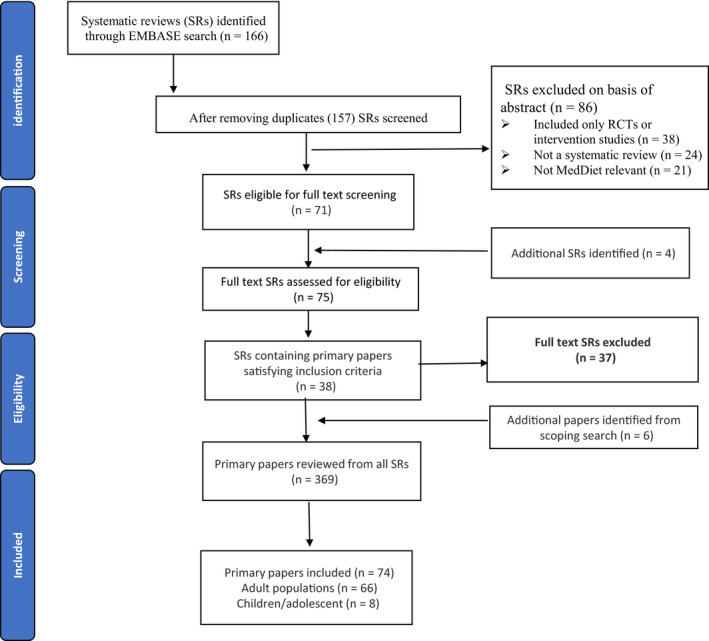
Flowchart illustrating the literature search and study selection process

The search identified 166 systematic reviews. However, after accounting for duplications 157 reviews on the MD published by 16 April 2018 and were eligible for screening. Abstracts were screened to ensure the articles complied with the inclusion criteria (Figure 1). Full texts of 75 systematic reviews, including four reviews identified through reference checking, were assessed and contained 37 papers that adhered to all aspects of the inclusion criteria. A total of 369 full‐text primary papers were reviewed from all included systematic reviews and 74 were accepted into this study, 66 in adults and eight in children.

### 3.1 Adult studies characteristics

We included 66 publications representing 87 group analyses, of these 54 analyses were related to data from the same 10 cohorts. These data were identified and grouped to ensure each cohort was only included once in the analysis (Table S1).

Thirty‐three publications presented combined data for males and females while 16 included both males and females but presented data separately. Fourteen publications related to all female cohorts while four publications reported on all male cohorts. Twenty‐eight publications included data on Mediterranean populations while 34 were from non‐Mediterranean regions. Full details on the characteristics of the publications included are outlined in (Table S2).

### 3.2 Variability in Mediterranean Diet scores

Most of the studies reported using the original, or a modified MDS, or the aMED (alternate Mediterranean Diet) score. The most commonly used scale to derive the scores was 0–9 which was used in 38/66 included papers (25/38 MDS and 14/38 aMED). Seven papers used a scale of 0–8, with five using aMED and two reporting the MDS. Where less than nine components were included, this was often due to the omission of dairy products or the combining of fruit and vegetables into the same food group. Details of the scores used are in included study characteristics (Table S2).

### 3.3 Adult intake data

Data on food groups, macronutrients, and micronutrients were extracted and are presented in (Tables [Link], [Link], [Link]). We had intended to analyze and explore the variability in all different categories where the foods formed part of the MD categorization. However, we were limited by inconsistency in reporting or categorization of the types of foods or nutrients for instance fat intake and intake of cereal foods were reported with considerable variability. We, therefore, analyzed data on: fruits and vegetables, meat, fish, and dairy from the food groups as the main components of MD. We also analyzed the available micronutrient data where there was a minimum of three studies reporting the same nutrient, after excluding duplicate publications from the same cohort. However, all data are reported in (Tables [Link], [Link], [Link]).

### 3.4 Food groups intake and variability

Data on olive oil, poultry, and nut intakes were very scarce while data on legumes and cereals were inconsistently reported. For example, while 12/66 papers (not excluding multiple publications from same cohort) reported data on any olive oil intake, the data varied from frequency per week to mean grams per day to percentage of energy intake accompanied by no report of total energy intake. Only 7/66 papers reported any poultry intake data, again with some of them reporting on the same cohort. For legumes, there was variation in how the data were reported with many reporting intake combined with nuts. As for cereals, studies varied in reporting the type of cereals with some studies not defining cereals while others only included unrefined or whole grains. To maximize the utilization of the available data and to allow the inclusion of the various measures reported (servings, grams, or other units), we calculated variability as the proportional difference between the highest and lowest reported quantiles of the MDS. We also reported the corresponding absolute intake value of the food or nutrient according to extreme quantiles. We analyzed variability of fruit, vegetables, fish, meat, and dairy intakes. These variability data were calculated for all studies, as well as grouped by Mediterranean versus non‐Mediterranean populations. (Only studies that reported data for Mediterranean or non‐Mediterranean populations were included in this analysis so studies that provided combined data for Mediterranean and non‐Mediterranean countries were excluded.) Variability of food group intake is presented in (Table 2), and intake data, as reported, are in (Table S3).

**TABLE 2:** Difference in food and nutrient intake according to adherence to the Mediterranean Diet in Mediterranean and non‐Mediterranean regions

Food or nutrient	Region	Publications (*n* =)	Mean difference in intake across included studies^a^	Range of the differences across quantiles for the individual studies^b^	Corresponding absolute values reported by the studies^c^
Fruit	All	36^d^	119%	37%, 311%	150 g/day, 240 g/day
	Mediterranean	14	89%	43%, 191%	109 g/day, 169 g/ day
	Non‐Mediterranean	20	146%	37%, 311%	150 g/day , 240 g/day
Vegetable	All	36	102%	32%, 196%	62 g/1,000 kcal, 75 g/day
	Mediterranean	14	91%	32%, 180%	62 g/1,000 kcal, 3.6 servings/week
	Non‐Mediterranean	19	112%	38%, 196%	71 g/day, 75 g/day
Fish	All	32	278%	31%, 4,400%	9 g/1,000 kcal, 0.88 servings/day
	Mediterranean	12	132%	35%, 300%	9 g/1,000 kcal, 0.45 servings/day
	Non‐Mediterranean	18	396%	65%, 4,400%	13 g/day, 0.88 servings/day
Meat	All	38	−23%	67%, −75%	1.4 servings/day, −57 g/day
	Mediterranean	14	−26%	1%, −50%	1 g/day, −0.2 servings/day
	Non‐Mediterranean	21	−23%	67%, −75%	1.4 servings/day, −57 g/day
Dairy	All	28	−31%	−72%, 114%	−13% energy, 2.4 servings/day
	Mediterranean	13	−41%	−72%, −13%	−13% energy, −0.3 servings/week
	Non‐Mediterranean	15	−24%	−67%, 114%	0.4 servings/day, 2.4 servings/day
Protein	All	18	1%	−14%, 1%	−2.5% energy, 12.3 g/day
	Mediterranean	8	−1%	−8%, −1%	−1.5% energy, 5.7 g/day
	Non‐Mediterranean	9	3%	−14%, 3%	−2.5% energy, 12.3 g/day
Calcium	All	9	1%	−20%, 30%	−254 mg/day, 173 mg/day
	Mediterranean	3	−5%	−13%, 8%	−119 mg/day, 100 mg/day
	Non‐Mediterranean	6	4%	−20%, 30%	−254 mg/day, 173 mg/day
Vitamin D	All	4	7%	0%, 10%	−0.02 µg/day, 0.18 µg/day
Magnesium	All	5	27%	9%, 48%	25.2 mg/day, 163 mg/day
Potassium	All	5	29%	8%, 29%	230 mg/day, 2,000 mg/day
Folate	All	10	35%	20%, 49%	54.6 µg/day, 193 µg/day
Vitamin C	All	12	65%	130%, 23%	229 mg/day, 20 mg/day

^a^ Values are the overall mean of the percentage difference in reported intake between the highest and lowest quantile of Mediterranean Diet adherence across all the included publications, see Tables [Link], [Link], [Link] However, as many cohort studies published data in more than 1 publication, specific studies were used as described in the methods section and in Table S1.

^b^ Values are the lowest and highest percentage difference reported from the included studies.

^c^ Values are the corresponding absolute values reported for the individual studies for the lowest and highest percentage differences reported across the included studies.

^d^ The contribution of the numbers of studies in the Mediterranean countries and non‐Mediterranean may not total “All studies” as some studies covered both categories.

Reported data were inspected, and studies were excluded from the individual analysis presented in Table 2 if they included unusable data or were duplicates for the same cohort. For example, data on fruit intake were reported in 47/66 included papers (63/87 analyses). After excluding studies with unusable data and accounting for multiple publications, 36/87 group analyses were included in the variability analysis. The final included number of studies for each food group varied from 28 for dairy intake to 38 for meat intake (Table 2).

**FIGURE 2:**
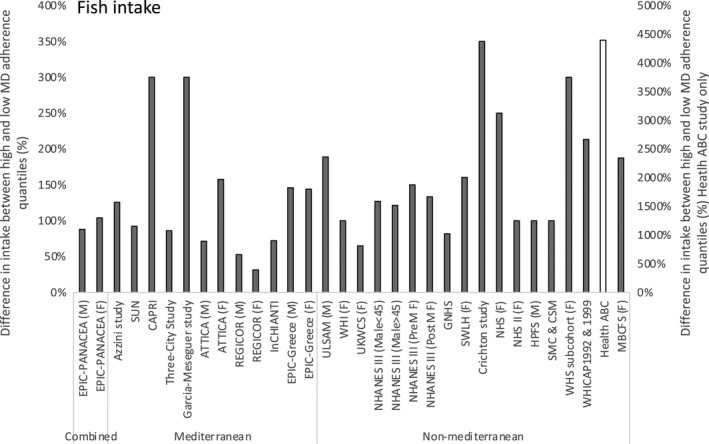
Difference in fish intake between participants in the highest and lowest quantile of Mediterranean Diet adherence by publication and region. Values are the percentage difference in reported intake between the highest and lowest quantile of Mediterranean Diet adherence ((Highest quantile–Lowest quantile)/ Lowest quantile)*100. M = Male, F = Female. If not specified, data are combined for males and females. Details of all publications of included studies are presented in (Table S2). However, details of the publications found in this and the following figures are below: Combined region publications: EPIC‐PANACEA (Romaguera et al., 2009). Mediterranean region publications; EPIC‐Spain (Buckland et al., 2009), EPIC (Buckland et al., 2011), Three City‐study (Feart, Alles, Merle, Samieri, & Barberger‐Gateau, 2012), Supplémentation en Vitamines et Minéraux AntioXydants (SU.VI.MAX) (Kesse‐Guyot et al., 2013), Seguimiento Universidad de Navarra Follow‐up Project (SUN) (Martinez‐Gonzalez et al., 2011), Registre Gironí del Cor [Girona Heart Registry] (REGICOR) (Schroder, Marrugat, Vila, Covas, & Elosua, 2004), Invecchiare in Chianti, aging in the Chianti area Study (InCHIANTI) (Talegawkar et al., 2012). Non‐Mediterranean region publications: Observation des Risques et de la Santé Cardiovasculaire au Luxembourg (ORISCAV‐LUX) (Alkerwi et al., 2015), Tehran Lipid and Glucose Study (TLGS) (Asghari et al., 2013), Uppsala Longitudinal Study of Adult Men (ULSAM) (Ax et al., 2014), Guangzhou Nutrition and Health Study (GNHS) (Chen et al., 2016), Swedish Women's Lifestyle and Health cohort study (SWLH) (Couto et al., 2013), Nurses Health Study (NHS) (Fung et al., 2009), Nurses Health Study II (NHS II) (Tobias et al., 2012), Cache County Study on Memory, Health and Aging (CCMS) (Wengreen et al., 2013) and REasons for Geographic and Racial Differences in Stroke (REGARDS) (Whalen et al., 2017)

Intake of food groups presumed to be beneficial Mediterranean dietary components (i.e., fruit, vegetables, and fish) varied greatly. For example, the overall average variability of fruit intake between extreme quantiles of the MDS across studies was 119%, with the range of this mean difference being 37% to 311%. For the corresponding absolute values reported for the study with a 37% difference in fruit intake across quantiles, the absolute difference was 150 g whereas that for 311% was 240 g, Table 2. However, for Mediterranean populations, the greatest difference between highest and lowest intake quantiles was equivalent to 169 g/day (equivalent to approximately 2.1, 80 g portions per day), (Table 2). The average overall variability between the highest and lowest quantiles was reported for the food group fish (278%). For fish, the greatest difference between the highest and lowest quantiles was equivalent to 0.88 serving/day and this variation in fish intake was smaller in studies from Mediterranean populations, corresponding to 0.45 serving/day (Figure 2). Variability of intake for food groups considered detrimental components in the MDP (namely meat and dairy products which were either scored negatively or were omitted during the construction of the score) ranged from −57 g/day to + 1.4 serving/ day for meat, and from an increase of 2.43 serving/day to a decrease of −13.3% energy for dairy. However, the difference of 1.4 serving came from the only study that defined a serving, which in this case was defined as one ounce (Shahar et al., 2012).

**FIGURE 3:**
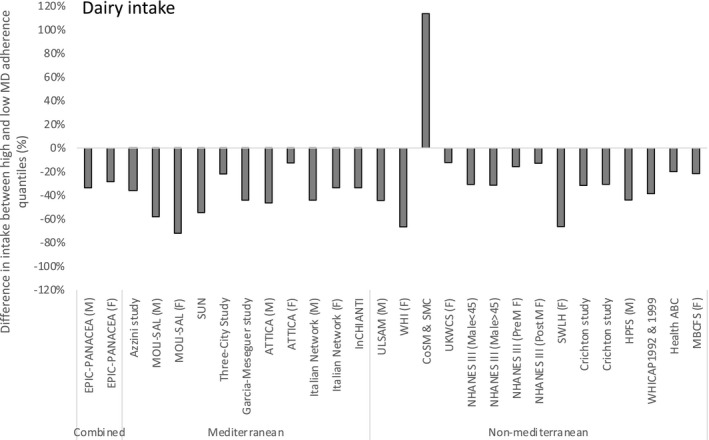
Difference in dairy intake between participants in the highest and lowest quantile of Mediterranean Diet adherence by publication and region. Values are the percentage difference in reported intake between the highest and lowest quantile of Mediterranean Diet adherence ((Highest quantile – Lowest quantile)/ Lowest quantile)*100. M = Male, F = Female. If not specified, data are combined for males and females. Details of all publications of included studies are presented in (Table S2)

Data from Mediterranean populations showed lower average intake in the highest quantile for dairy (−41% versus −24%; Figure 3) but not for meat (−26% versus −23%; Table 2). However, reporting for the dairy food group data varied considerably. While most studies reported intake of dairy products without specifying the type, some studies specified that the type of dairy products included and these were either fermented, full fat, or including milk.

### 3.5 Macronutrient intake and variability

Data on various macronutrients (protein, fat, and carbohydrate) were less commonly reported than food groups. We were unable to analyze data on fat intake since studies reported intake in too many ways to allow comparison. Some studies reported total fat intake while other reported it as either saturated or unsaturated fats, with the latter being reported as either poly‐ or monounsaturated fats.

Although data on protein intake were not reported by many studies, reporting was more consistent than with fat intake, and we analyzed the available data to complement data on meat intake since meat is considered a detrimental component of a Mediterranean dietary pattern. See Table 2 and Table S4.

**FIGURE 4:**
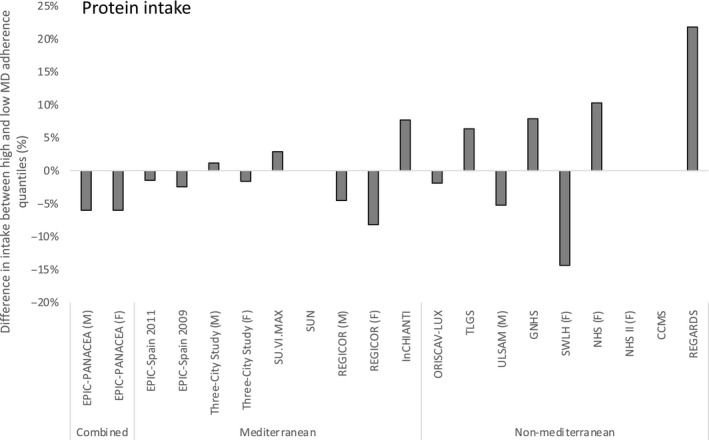
Difference in protein intake between participants in the highest and lowest quantile of Mediterranean Diet adherence by publication and region. Values are the percentage difference in reported intake between the highest and lowest quantile of Mediterranean Diet adherence ((Highest quantile – Lowest quantile)/ Lowest quantile)*100. M = Male, F = Female. If not specified, data are combined for males and females. Details of all publications of included studies are presented in (Table S2)

Variability of protein intake between those considered as highest and lowest adherers to MDP ranged from an increase of 12.3 g/day, to a decrease of 2.5% Energy intake. However, this variability was less prominent in Mediterranean populations ranging from 5.7 g/day to −1.5% of total energy intake (Figure 4).

### 3.6 Micronutrient variability

Overall, very few studies included intakes of the micronutrient vitamins and minerals. Vitamin C, folate, and calcium were the most frequently reported in 15, 11, and 10 group analyses, respectively. Selenium was only reported in one, iron intake in two, and no included studies reported on iodine intake.

Due to limited data, only calcium intake was analyzed by region. Overall, the average difference in calcium intake was 1% ranging from an increase of 173 mg/day to −254 mg/day. This was less marked in Mediterranean regions, ranging from a difference between the highest and lowest quantile of 100 mg/day more to 119 mg/day less. The greatest average variation was reported for vitamin C intake with the intake in the highest versus lowest quantiles varying from 20 to 229 mg/day, resulting in a mean proportional difference of 65% across quantiles, see Table 2 and Table S5.

### 3.7 Dietary assessment method

We had expected to analyze variability of the MDS by grouping according to the type of dietary assessment method, that is, food frequency methods (FFQs) versus “record methods” such as 24‐hr recalls, however, ~90% of the methods reported were based on FFQs so analysis by dietary assessment method was not possible (Table S2).

### 3.8 Characteristics of childhood studies

We identified eight studies that reported on Mediterranean Diet in children and adolescents (Grosso et al., 2013; Kontogianni et al., 2008; Lazarou, Panagiotakos, & Matalas, 2009; Magriplis et al., 2011; Monjardino, Lucas, Ramos, & Barros, 2012; Noale et al., 2014; Schröder, Mendez, Ribas‐Barba, Covas, & Serra‐Majem, 2010; Serra‐Majem, Ribas, García, Pérez‐Rodrigo, & Aranceta, 2003). One of the included studies (Lazarou et al., 2009) reported data that could not be used as it was categorized by frequency of intake for each food group (Lazarou et al., 2009). Apart from Monjardino et al. (2012), which was a cohort study, the remaining seven studies were of cross‐sectional design. All the included studies used the KIDMED score, except Noale et al. (2014), that included children 12–19 years old and used the MDS scoring system. Serra‐Majem et al. (2003) included participants from 6–24 years old and presented their data separately in four different categories by gender for two age subgroups of 6–14 and 15–24 years. All the included studies were in children from Mediterranean countries with sample sizes ranging from 552 to 4,580 participants. Only two studies reported any food group intake data (Grosso et al., 2013; Schröder et al., 2010) with sweets, fruit, vegetables, fish, and meat being the only food groups reported by both studies. Four studies reported any macronutrient data with two of them reporting this as percentage energy. One study reported only calcium intake. Data from studies in children were not analyzed but are presented in (Tables [Link], [Link], [Link]).

## 4 DISCUSSION

The current study has shown inconsistencies in both definitions and construction of the MDS which translate into large variability in food and nutrient intake according to the MD adherence. These findings suggest that the Mediterranean Diet is an inconsistent dietary pattern with both within‐ and between‐country variation. Only 20% of the studies we searched for provided data for the food group or nutrient composition across the MD distribution, with no included study reporting composition for all the dietary components. The types of dietary intakes reported varied greatly with some food groups only reported in 10%–15% of studies (e.g., olive oil and poultry), whereas others were reported in 70% (e.g., fruit and vegetables). For macronutrients and micronutrients, respectively, between 25%–35% and 0%–17% of studies reported on these nutrients. In general, there was greater variability in food intakes in non‐Mediterranean regions compared to Mediterranean regions. For meat and dairy components, there were inconsistencies in how they were incorporated into the scores (either positively or negatively scored), and therefore, better adherence to a MDP may translate to either higher or lower intakes of these foods with subsequent impact on the protein intakes reported in our study. Data on MDP in children were very limited preventing analysis or conclusions.

We expected to be able to capture food and nutrient intakes by MDP adherence but due to the lack of consistency with both derivation of scores, and reporting of foods and nutrients as well as the various distribution categories reported, we were only able to capture this variability by expressing the proportion of the differences across the studies. We recognize this as a limitation of our study. We were also limited by the relatively small number of studies that provided data on food and, particularly, nutrient intake in relation to the distribution of the MDS. We recommend that future studies on the MD and health outcomes report intakes of both foods and nutrients by adherence to the MDS, in order to allow for more detailed comparison of the composition and variation of the MD within and between countries.

We also expected to find variability between studies using different methods of dietary assessment and planned to determine whether variability was also due to type of dietary assessment method, for instance by stratifying the data; however, as ~90% of studies used food frequency questionnaires, we were unable to make this comparison. Since the majority of studies were in FFQs, this demonstrates that variability across categories of adherence to the MD is more likely due to other factors such as differences in the construction of the MDS according to the different types of MDS and study, country and regional specific distributions of the food and nutrient components used to derive the MDS.

We found that the definition of food groups and nutrients were not consistent between studies that had referenced the same MDS. For example, the “vegetable food group” sometimes included legumes or potatoes or other starchy vegetables. Similarly, meat may have included meat products or processed meats or poultry as well as eggs, whereas dairy may be classified as total dairy, whole fat dairy, or without cheese (Panagiotakos, Pitsavos, & Stefanadis, 2006; Toledo et al., 2013). Recent studies have also found that MD indexes vary in their construction and consequently how these classify the adherence of individuals (Hernandez‐Ruiz et al., 2018; Olmedo‐Requena et al., 2019). However, to our knowledge this is the first study to review and capture the variability in reporting of the MD in terms of its construction and impact on foods and nutrient intakes across different countries.

While studies have shown a protective effect of consuming a diet more closely aligned to the MDP for noncommunicable disease outcomes (Koloverou et al., 2014; Liyanage et al., 2016), the variability in the MDS which we have identified from the limited data reported by investigators means that it is difficult to translate this dietary pattern with any degree of consistency for dietary guidelines and health policy in different settings, countries, and regions. This is important as to develop dietary recommendations the amounts of foods or nutrients required by different sectors of the population need to be calculated. This greater understanding of the constituent components of the MDP is particularly important for non‐Mediterranean regions of the world where the types of food consumed within the categories used to construct the score, for example, for vegetables may differ from Mediterranean regions. It is also important to understand the MD eating pattern more fully as health policy and guidelines have a direct impact on agricultural production practices.

Within the findings of a protective effect of the MDP for noncommunicable diseases, closer inspection indicates some contradictory findings in relation to musculoskeletal conditions (osteoporosis, risk of fracture or sarcopenia; Craig et al., 2017). This is likely to be due to a lack of clarity on whether the MDP confers sufficient quantities of the nutrients calcium and protein required for protection of bone or skeletal muscle health. We also found insufficient data for reported calcium intake to make comparisons with nutritional guidelines for musculoskeletal health, as we had originally planned. Moreover, at this stage we do not know whether adherence to the MDP would impact on growth and on bone mineralization in children and adolescents. We would need RCTs to determine the effect of the MDP on muscle and bone health at all ages(Craig et al., 2017).

Due to the limitations of the data provided in previous studies and to the variation in methods of reporting of the MDS, we recommend that reporting of the MDS should be changed for all future studies (Paterson Katherine et al., 2018). Future studies should include and detail not only the food group and nutrient consumption across the distribution of the MDP but also the derivation of the components of the MDP. For instance, noting whether the “fruit” category includes nut consumption or not or whether the “vegetable” category includes potatoes. This would aid researchers and policy makers in interpreting the results of future studies and identifying areas of consistency and inconsistency. Better description and reporting of the components of the MDP, that is, the quantities of foods and nutrients would also help researchers to understand the benefits of the MDP on chronic diseases better and to know how to translate these benefits within countries into healthy eating guidelines as well as clinical advice. This would also enable more targeted population approaches to improve the health of populations of both adults and children in relation to consuming the Mediterranean Diet. Moreover, although the current MDS do not use absolute values for cut points for the categories of foods within their construction, future consideration could be given to developing an a priori set of cut points to be used in studies rather than relying on individual study or country‐specific food or nutrient distributions for construction of the MDS.

In conclusion, we found few studies reported consumption of foods and nutrients across the MDP in adults, with even fewer in children. We also found considerable variability in the foods and nutrients that were reported which makes interpretation of this pattern difficult for translation into health policy and dietary guidelines. As steps need to be taken to improve assessment and reporting of components of the MD, we recommend that all future studies should report the full range of foods and nutrients consumed across the Mediterranean dietary pattern.

## CONFLICT OF INTEREST

The authors have no conflicts of interest.

## TRANSPARENCY DECLARATION

The lead author affirms that this manuscript is an honest, accurate, and transparent account of the study being reported. The reporting of this work is compliant with PRISMA guidelines. The lead author affirms that no important aspects of the study have been omitted and that any discrepancies from the study as planned have been explained.

## Supporting information

Table S1

Table S2

Table S3

Table S4

Table S5

Table S6

Table S7

Table S8

Table S9
