# Supplementary material for: High variability of food and nutrient intake exists across the Mediterranean Dietary Pattern—A systematic review
Source: Food Sci Nutr. 2020 Jul 29;8(9):4907–18. doi: 10.1002/fsn3.1784 (PMC7500794; doi:10.1002/fsn3.1784)
Supplement: Supplementary file 1 — Table S1 [file FSN3-8-4907-s001.docx]

**Supplementary table 1: Identification of data for analyses in cohorts with multiple publications**

| **Cohort** | **Publications identified** | **Publications used in analyses** | | |
| --- | --- | --- | --- | --- |
|  |  | **Food groups** | **Macronutrient** | **Micronutrient** |
| **European Prospective Investigation into Cancer (EPIC)** | Romaguera *et al.*, 2009 (1) | x | Protein |  |
|  | Agnoli *et al.*, 2013 (2) |  |  |  |
|  | Buckland *et al.*, 2013 (3) |  |  |  |
|  | Buckland *et al.*, 2014 (4) | Dairy |  |  |
|  | Molina-Montes *et al.*, 2017 (5) |  | x | x |
| **EPIC Greece** | Rossi *et al.*, 2013 (6) |  | x |  |
|  | Trichopoulou *et al.*, 2005 (7) | x | Energy and carbohydrate |  |
| **EPIC Spain** | Mendez *et al.*, 2006 (8) |  |  |  |
|  | Buckland *et al.*, 2009 (9) |  | x | x |
|  | Buckland *et al.*, 2011 (10) |  |  |  |
| **Attica Study** | Chrysohoou *et al.*, 2004 (11) |  |  |  |
|  | Pitsavos *et al.*, 2005 (12) |  |  |  |
|  | Panagiotakos *et al.*, 2006 (13) | x | x |  |
| **Women's Health Initiative** | Bertoria *et al.*, 2014 (14) | x |  |  |
|  | Vargas *et al.*, 2016 (15) | Cereals | x |  |
| **Seguimiento Universidad de Navarra Follow-up [SUN] Project** | Sanchez-Villegas *et al.*, 2006 (16) |  |  |  |
|  | Martinez-Gonzalez *et al.*, 2008 (17) | Fruit |  |  |
|  | Nunez-Cordoba *et al.*, 2009 (18) |  |  | Mg, K, Ca |
|  | Sanchez-Villegas *et al.*, 2009 (19) |  |  |  |
|  | Beunza *et al.*, 2010 (20) |  |  | Sodium |
|  | Martinez-Gonzalez *et al.*, 2011 (21) | Olive oil | x | Vitamin C |
|  | Dominguez *et al.*, 2013 (22) | x |  |  |
|  | Galbete *et al.*, 2015 (23) |  |  |  |
| **Swedish Mammography**  **Cohort and Cohort of Swedish Men** | Tektonidis *et al.*, 2015 (24) | Females |  |  |
|  | Byberg *et al.*, 2016 (25) | Dairy and cereals | x | x |
|  | Tektonidis *et al.*, 2016 (26) | Males |  |  |
|  | Larsson *et al.*, 2017 (27) | Combined data |  |  |
| **Three-City Study** | Feart *et al.*, 2009 (28) |  |  |  |
|  | Feart *et al.*, 2012 (29) | x | x |  |
| **Health Professionals Follow-up study** | de Koning *et al.*, 2011 (30) |  |  |  |
|  | Fung *et al.*, 2010 (31) |  | x | Ca, folate |
|  | Gao *et al.*, 2007 (32) |  | Energy and alcohol | Vitamin C |
|  | Kenfield *et al.*, 2014 (33) | x |  |  |
|  | Lopez-Garcia *et al.*, 2014 (34) |  |  |  |
|  | Crous-Bou *et al.*, 2014 (35) |  |  |  |
| **Nurses Health Study** | Mantzoros *et al.*, 2006 (36) |  |  |  |
|  | Gao *et al.*, 2007 (32) |  |  | Vitamin C |
|  | Fung *et al.*, 2009 (37) |  | x |  |
|  | Fung *et al.*, 2010 (31) |  | PUFA | Ca, folate |
|  | Samieri *et al.*, 2013 (38) |  |  |  |
|  | Lopez-Garcia *et al.*, 2014 (34) |  |  |  |
|  | Hu *et al.*, 2015 (39) | x |  |  |
| **Nurses Health Study II** | Tobias *et al.*, 2012 (40) |  | x |  |
|  | Hu *et al.*, 2015 (39) | x |  |  |

x indicates the publication that was used in the analyses, where data were available for multiple publications from the same cohort the report with the largest sample size was used. In cases where additional foods or nutrients were available in other publications these are indicated.

**References**

1. Romaguera D, Norat T, Mouw T, May AM, Bamia C, Slimani N, Travier N, Besson H, Luan J, Wareham N, et al. Adherence to the Mediterranean diet is associated with lower abdominal adiposity in European men and women. J Nutr 2009;139:1728-37.

2. Agnoli C, Grioni S, Sieri S, Palli D, Masala G, Sacerdote C, Vineis P, Tumino R, Giurdanella MC, Pala V, et al. Italian Mediterranean Index and risk of colorectal cancer in the Italian section of the EPIC cohort. Int J Cancer 2013;132:1404-11.

3. Buckland G, Travier N, Cottet V, Gonzalez CA, Lujan-Barroso L, Agudo A, Trichopoulou A, Lagiou P, Trichopoulos D, Peeters PH, et al. Adherence to the mediterranean diet and risk of breast cancer in the European prospective investigation into cancer and nutrition cohort study. Int J Cancer 2013;132:2918-27.

4. Buckland G, Ros MM, Roswall N, Bueno-de-Mesquita HB, Travier N, Tjonneland A, Kiemeney LA, Sacerdote C, Tumino R, Ljungberg B, et al. Adherence to the Mediterranean diet and risk of bladder cancer in the EPIC cohort study. Int J Cancer 2014;134:2504-11.

5. Molina-Montes E, Sanchez MJ, Buckland G, Bueno-de-Mesquita HB, Weiderpass E, Amiano P, Wark PA, Kuhn T, Katzke V, Huerta JM, et al. Mediterranean diet and risk of pancreatic cancer in the European Prospective Investigation into Cancer and Nutrition cohort. Br J Cancer 2017;116:811-820.

6. Rossi M, Turati F, Lagiou P, Trichopoulos D, Augustin LS, La Vecchia C, Trichopoulou A. Mediterranean diet and glycaemic load in relation to incidence of type 2 diabetes: results from the Greek cohort of the population-based European Prospective Investigation into Cancer and Nutrition (EPIC). Diabetologia 2013;56:2405-13.

7. Trichopoulou A, Naska A, Orfanos P, Trichopoulos D. Mediterranean diet in relation to body mass index and waist-to-hip ratio: the Greek European Prospective Investigation into Cancer and Nutrition Study. Am J Clin Nutr 2005;82:935-40.

8. Mendez MA, Popkin BM, Jakszyn P, Berenguer A, Tormo MJ, Sanchez MJ, Quiros JR, Pera G, Navarro C, Martinez C, et al. Adherence to a Mediterranean diet is associated with reduced 3-year incidence of obesity. J Nutr 2006;136:2934-8.

9. Buckland G, Gonzalez CA, Agudo A, Vilardell M, Berenguer A, Amiano P, Ardanaz E, Arriola L, Barricarte A, Basterretxea M, et al. Adherence to the Mediterranean diet and risk of coronary heart disease in the Spanish EPIC Cohort Study. Am J Epidemiol 2009;170:1518-29.

10. Buckland G, Agudo A, Travier N, Huerta JM, Cirera L, Tormo MJ, Navarro C, Chirlaque MD, Moreno-Iribas C, Ardanaz E, et al. Adherence to the Mediterranean diet reduces mortality in the Spanish cohort of the European Prospective Investigation into Cancer and Nutrition (EPIC-Spain). Br J Nutr 2011;106:1581-91.

11. Chrysohoou C, Panagiotakos DB, Pitsavos C, Das UN, Stefanadis C. Adherence to the Mediterranean diet attenuates inflammation and coagulation process in healthy adults: The ATTICA Study. J Am Coll Cardiol 2004;44:152-8.

12. Pitsavos C, Panagiotakos DB, Tzima N, Chrysohoou C, Economou M, Zampelas A, Stefanadis C. Adherence to the Mediterranean diet is associated with total antioxidant capacity in healthy adults: the ATTICA study. Am J Clin Nutr 2005;82:694-9.

13. Panagiotakos DB, Pitsavos C, Stefanadis C. Dietary patterns: a Mediterranean diet score and its relation to clinical and biological markers of cardiovascular disease risk. Nutr Metab Cardiovasc Dis 2006;16:559-68.

14. Bertoia ML, Triche EW, Michaud DS, Baylin A, Hogan JW, Neuhouser ML, Tinker LF, Van Horn L, Waring ME, Li W, et al. Mediterranean and Dietary Approaches to Stop Hypertension dietary patterns and risk of sudden cardiac death in postmenopausal women. Am J Clin Nutr 2014;99:344-51.

15. Vargas AJ, Neuhouser ML, George SM, Thomson CA, Ho GY, Rohan TE, Kato I, Nassir R, Hou L, Manson JE. Diet Quality and Colorectal Cancer Risk in the Women's Health Initiative Observational Study. Am J Epidemiol 2016;184:23-32.

16. Sanchez-Villegas A, Bes-Rastrollo M, Martinez-Gonzalez MA, Serra-Majem L. Adherence to a Mediterranean dietary pattern and weight gain in a follow-up study: the SUN cohort. Int J Obes (Lond) 2006;30:350-8.

17. Martinez-Gonzalez MA, de la Fuente-Arrillaga C, Nunez-Cordoba JM, Basterra-Gortari FJ, Beunza JJ, Vazquez Z, Benito S, Tortosa A, Bes-Rastrollo M. Adherence to Mediterranean diet and risk of developing diabetes: prospective cohort study. Bmj 2008;336:1348-51.

18. Núñez-Córdoba JM, Valencia-Serrano F, Toledo E, Alonso A, Martínez-González MA. The Mediterranean diet and incidence of hypertension: The Seguimiento Universidad de Navarra (SUN) study. American Journal of Epidemiology 2009;169:339-346.

19. Sanchez-Villegas A, Delgado-Rodriguez M, Alonso A, Schlatter J, Lahortiga F, Serra Majem L, Martinez-Gonzalez MA. Association of the Mediterranean dietary pattern with the incidence of depression: the Seguimiento Universidad de Navarra/University of Navarra follow-up (SUN) cohort. Arch Gen Psychiatry 2009;66:1090-8.

20. Beunza JJ, Toledo E, Hu FB, Bes-Rastrollo M, Serrano-Martinez M, Sanchez-Villegas A, Martinez JA, Martinez-Gonzalez MA. Adherence to the Mediterranean diet, long-term weight change, and incident overweight or obesity: the Seguimiento Universidad de Navarra (SUN) cohort. Am J Clin Nutr 2010;92:1484-93.

21. Martinez-Gonzalez MA, Garcia-Lopez M, Bes-Rastrollo M, Toledo E, Martinez-Lapiscina EH, Delgado-Rodriguez M, Vazquez Z, Benito S, Beunza JJ. Mediterranean diet and the incidence of cardiovascular disease: a Spanish cohort. Nutr Metab Cardiovasc Dis 2011;21:237-44.

22. Dominguez LJ, Bes-Rastrollo M, de la Fuente-Arrillaga C, Toledo E, Beunza JJ, Barbagallo M, Martinez-Gonzalez MA. Similar prediction of total mortality, diabetes incidence and cardiovascular events using relative- and absolute-component Mediterranean diet score: the SUN cohort. Nutr Metab Cardiovasc Dis 2013;23:451-8.

23. Galbete C, Toledo E, Toledo JB, Bes-Rastrollo M, Buil-Cosiales P, Marti A, Guillen-Grima F, Martinez-Gonzalez MA. Mediterranean diet and cognitive function: the SUN project. J Nutr Health Aging 2015;19:305-12.

24. Tektonidis TG, Akesson A, Gigante B, Wolk A, Larsson SC. A Mediterranean diet and risk of myocardial infarction, heart failure and stroke: A population-based cohort study. Atherosclerosis 2015;243:93-8.

25. Byberg L, Bellavia A, Larsson SC, Orsini N, Wolk A, Michaëlsson K. Mediterranean Diet and Hip Fracture in Swedish Men and Women. Journal of Bone and Mineral Research 2016;31:2098-2105.

26. Tektonidis TG, Akesson A, Gigante B, Wolk A, Larsson SC. Adherence to a Mediterranean diet is associated with reduced risk of heart failure in men. Eur J Heart Fail 2016;18:253-9.

27. Larsson SC, Hakansson N, Wolk A. Healthy dietary patterns and incidence of biliary tract and gallbladder cancer in a prospective study of women and men. Eur J Cancer 2017;70:42-47.

28. Feart C, Samieri C, Rondeau V, Amieva H, Portet F, Dartigues JF, Scarmeas N, Barberger-Gateau P. Adherence to a Mediterranean diet, cognitive decline, and risk of dementia. Jama 2009;302:638-48.

29. Feart C, Alles B, Merle B, Samieri C, Barberger-Gateau P. Adherence to a Mediterranean diet and energy, macro-, and micronutrient intakes in older persons. J Physiol Biochem 2012;68:691-700.

30. de Koning L, Chiuve SE, Fung TT, Willett WC, Rimm EB, Hu FB. Diet-quality scores and the risk of type 2 diabetes in men. Diabetes Care 2011;34:1150-6.

31. Fung TT, Hu FB, Wu K, Chiuve SE, Fuchs CS, Giovannucci E. The Mediterranean and Dietary Approaches to Stop Hypertension (DASH) diets and colorectal cancer. Am J Clin Nutr 2010;92:1429-35.

32. Gao X, Chen H, Fung TT, Logroscino G, Schwarzschild MA, Hu FB, Ascherio A. Prospective study of dietary pattern and risk of Parkinson disease. Am J Clin Nutr 2007;86:1486-94.

33. Kenfield SA, DuPre N, Richman EL, Stampfer MJ, Chan JM, Giovannucci EL. Mediterranean diet and prostate cancer risk and mortality in the Health Professionals Follow-up Study. Eur Urol 2014;65:887-94.

34. Lopez-Garcia E, Rodriguez-Artalejo F, Li TY, Fung TT, Li S, Willett WC, Rimm EB, Hu FB. The Mediterranean-style dietary pattern and mortality among men and women with cardiovascular disease. Am J Clin Nutr 2014;99:172-80.

35. Crous-Bou M, Fung TT, Prescott J, Julin B, Du M, Sun Q, Rexrode KM, Hu FB, De Vivo I. Mediterranean diet and telomere length in Nurses’ Health Study: population based cohort study. BMJ : British Medical Journal 2014;349.

36. Mantzoros CS, Williams CJ, Manson JE, Meigs JB, Hu FB. Adherence to the Mediterranean dietary pattern is positively associated with plasma adiponectin concentrations in diabetic women. Am J Clin Nutr 2006;84:328-35.

37. Fung TT, Rexrode KM, Mantzoros CS, Manson JE, Willett WC, Hu FB. Mediterranean diet and incidence of and mortality from coronary heart disease and stroke in women. Circulation 2009;119:1093-100.

38. Samieri C, Okereke OI, E ED, Grodstein F. Long-term adherence to the Mediterranean diet is associated with overall cognitive status, but not cognitive decline, in women. J Nutr 2013;143:493-9.

39. Hu Y, Costenbader KH, Gao X, Hu FB, Karlson EW, Lu B. Mediterranean Diet and Incidence of Rheumatoid Arthritis in Women. Arthritis Care & Research 2015;67:597-606.

40. Tobias DK, Hu FB, Chavarro J, Rosner B, Mozaffarian D, Zhang C. Healthful dietary patterns and type 2 diabetes mellitus risk among women with a history of gestational diabetes mellitus. Archives of Internal Medicine 2012;172:1566-1572.
