# Supplementary material for: High variability of food and nutrient intake exists across the Mediterranean Dietary Pattern—A systematic review
Source: Food Sci Nutr. 2020 Jul 29;8(9):4907–18. doi: 10.1002/fsn3.1784 (PMC7500794; doi:10.1002/fsn3.1784)
Supplement: Supplementary file 2 — Table S2 [file FSN3-8-4907-s002.pdf]

Characteristics of included adult studies

Number	Cohort name	Author, year	Dates assessed	Scoring system	Score range	Original reference for score	Dietary assessment method	n	Sex	% Male (if combined data)	Age (y)	Location	Quantile used for reporting data	Metric used for reporting data	Nutrient data available	Reference
1	EPIC-Italy	Agnoli *et al.*, 2013	1993-1998	Italian Mediterranean Index score	1-11	Trichopoulou et al. N Engl J Med 2003; 348: 2599–608	SFFQ	45275	M&F	31.4	50 (mean)	Italy	Tertiles	Range	Alcohol, Food groups	Agnoli C, Grioni S, Sieri S, Palli D, Masala G, Sacerdote C, Vineis P, Tumino R, Giurdanella MC, Pala V, et al. Italian Mediterranean Index and risk of colorectal cancer in the Italian section of the EPIC cohort. Int J Cancer 2013;132:1404-11.
2	Observation of Cardiovascular Risk Factors in Luxembourg (ORISCAV-LUX)	Alkerwi *et al.* , 2014	2007-2009	MDS [median cut-points]	0-9	Trichopoulou et al. N Engl J Med 2003; 348: 2599–608	SFFQ	1352	M&F	Not reported	42.1 (Q1 mean), 46.4 (Q4 mean), 18-69 (range)	Luxembourg	Quartiles	Median	Alcohol, Macronutrients	Alkerwi A, Vernier C, Crichton GE, Sauvageot N, Shivappa N, Hebert JR. Cross-comparison of diet quality indices for predicting chronic disease risk: findings from the Observation of Cardiovascular Risk Factors in Luxembourg (ORISCAV-LUX) study. Br J Nutr 2015;113(2):259-69
3	Tehran Lipid and Glucose Study (TLGS)	Asgarhi *et al.* , 2013	1999-2001	MDS [median cut-points]	0-10	Trichopoulou et al. N Engl J Med 2003; 348: 2599–608	24h recall	469	M&F	43 (Q1), 49.6 (Q2), 44.8 (Q3), 46.5 (Q4)	38.7 (mean)	Tehran, Iran	Quartiles	Median	Macronutrients	Asghari G, Mirmiran P, Hosseni-Esfahani F, Nazeri P, Mehran M, Azizi F. Dietary quality among Tehranian adults in relation to lipid profile: findings from the Tehran Lipid and Glucose Study. J Health Popul Nutr 2013;31:37-48
4	Uppsala Longitudinal Study of Adult Men (ULSAM)	Ax *et al.* , 2014	1991-1995	Modified Mediterranean diet (mMED)	0-8	Trichopoulou et al. N Engl J Med 2003; 348: 2599–608	Food diary (7 day)	1044	M	-	71 (mean)	Sweden	Tertiles	Median	Macronutrients, Micronutrients, Food groups	Ax E, Garmo H, Grundmark B, Bill-Axelson A, Holmberg L, Becker W, Zethelius B, Cederholm T, Sjogren P. Dietary patterns and prostate cancer risk: report from the population based ULSAM cohort study of Swedish men. Nutr Cancer 2014:66(1):77-87
5	Azzini study	Azzini *et al.*, 2011	Not reported	MDS [median cut-points]	0-9	Trichopoulou et al. N Engl J Med 2003; 348: 2599–608	Food diary (4 day)	131	M&F	48.9	20-40	S. and Central Italy	Tertiles	Mean	Alcohol, Macronutrients, Food groups	Azzini E, Polito A, Fumagalli A, Intorre F, Venneria E, Durazzo A, Zaccaria M, Ciarapica D, Foddai MS, Mauro B, et al. Mediterranean Diet Effect: an Italian picture. Nutr J 2011;10:125
6	Women's Health Initiative (WHI)	Bertoia *et al.*, 2014	1993 & 1998	MDS [quintile cut-points]	0-40	Trichopoulou et al. N Engl J Med 2003; 348: 2599–608	SFFQ	93122	F	-	50-79	40 centres, USA	Quintiles	Mean	Food groups	Bertoia ML, Triche EW, Michaud DS, Baylin A, Hogan JW, Neuhouser ML, Tinker LF, Van Horn L, Waring ME, Li W, et al. Mediterranean and Dietary Approaches to Stop Hypertension dietary patterns and risk of sudden cardiac death in postmenopausal women. Am J Clin Nutr 2014;99(2):344-51
7	Seguimiento Universidad de Navarra Follow-up Project (SUN)	Beunza *et al.*, 2010	1999-2007	MDS [median cut-points]	0-9	Trichopoulou et al. N Engl J Med 2003; 348: 2599–608	SFFQ	10376	M&F	46	38 (mean)	Spain	Tertiles	Mean	Alcohol, Micronutrients,	Beunza JJ, Toledo E, Hu FB, Bes-Rastrollo M, Serrano-Martinez M, Sanchez-Villegas A, Martinez JA, Martinez-Gonzalez MA. Adherence to the Mediterranean diet, long-term weight change, and incident overweight or obesity: the Seguimiento Universidad de Navarra (SUN) cohort. Am J Clin Nutr 2010;92:1484-93.
8	EPIC-Spain	Buckland *et al.*, 2011	1992-1996	Relative MDS (rMED)	0-18	Trichopoulou et al. N Engl J Med 2003; 348: 2599–608	Dietary questionaire	40622	M&F	37.7	29-69	Spain	Tertiles	Median except for Energy	Macronutrients	Buckland G, Agudo A, Travier N, Huerta JM, Cirera L, Tormo MJ, Navarro C, Chirlaque MD, Moreno-Iribas C, Ardanaz E, et al. Adherence to the Mediterranean diet reduces mortality in the Spanish cohort of the European Prospective Investigation into Cancer and Nutrition (EPIC-Spain). Br J Nutr 2011;106:1581-91.
9	EPIC-Spain	Buckland *et al.*, 2009	1992-1996	Relative MDS (rMED)	0-18	Trichopoulou et al. N Engl J Med 2003; 348: 2599–608	Dietary questionaire	41438	M&F	37.6	29-69	Spain	Tertiles	Median	Macronutrients, Micronutrients,	Buckland G, Gonzalez CA, Agudo A, Vilardell M, Berenguer A, Amiano P, Ardanaz E, Arriola L, Barricarte A, Basterretxea M, et al. Adherence to the Mediterranean diet and risk of coronary heart disease in the Spanish EPIC Cohort Study. Am J Epidemiol 2009;170:1518-29.
10	EPIC	Buckland *et al.* , 2014	1991-2000	Relative MDS (rMED)	0-18	Trichopoulou et al. N Engl J Med 2003; 348: 2599–608	Country-specific	477312	M&F	29.8	51.2 (mean)	10 European countries	Tertiles	Mean	Food groups	Buckland G, Ros MM, Roswall N, Bueno-de-Mesquita HB, Travier N, Tjonneland A, Kiemeney LA, Sacerdote C, Tumino R, Ljungberg B, et al. Adherence to the Mediterranean diet and risk of bladder cancer in the EPIC cohort study. Int J Cancer 2014;134:2504-11.
11	EPIC	Buckland *et al.*, 2013	1992-2000	Adapted Relative MDS (arMED)	0-16	Trichopoulou et al. N Engl J Med 2003; 348: 2599–608	Country-specific	335062	F	-	50.8 (mean)	10 European countries	Tertiles	Mean	Alcohol, Macronutrients	Buckland G, Travier N, Cottet V, Gonzalez CA, Lujan-Barroso L, Agudo A, Trichopoulou A, Lagiou P, Trichopoulos D, Peeters PH, et al. Adherence to the mediterranean diet and risk of breast cancer in the European prospective investigation into cancer and nutrition cohort study. Int J Cancer 2013;132:2918-27.
12	Cohort of Swedish Men (CoSM) and Swedish Mammography Cohort (SMC)	Byberg *et al* .,2016	1997	Modified Mediterranean diet (mMED)	0-8	Trichopoulou et al. N Engl J Med 2003; 348: 2599–608	FFQ	71333	M&F	53	60.5 (T1 mean), 59.8 (T3 mean)	Sweden	Tertiles	Median	Alcohol, Micronutrients, Food groups	Byberg L, Bellavia A, Larsson SC, Orsini N, Wolk A, Michaëlsson K. Mediterranean Diet and Hip Fracture in Swedish Men and Women. Journal of Bone and Mineral Research 2016;31:2098-2105.
13	The UK Women's Cohort Study (UKWCS)	Cade *et al.* , 2011	1995-1998	MDS [median cut-points]	0-10	Trichopoulou et al. N Engl J Med 2003; 348: 2599–608	FFQ	33731	F	-	52 (mean), 35-69 (range)	UK	sextile	Mean	Macronutrients, Micronutrients, Food groups	Cade JE, Taylor EF, Burley VJ, Greenwood DC. Does the Mediterranean dietary pattern or the Healthy Diet Index influence the risk of breast cancer in a large British cohort of women? Eur J Clin Nutr 2011;65(8):920-8
14	NHANES III	Carter *et al.* , 2010	1988-94	MDS-Attica	0-55	Panagiotakos et al., Nutr Metab Cardiovasc Dis 2006;16:559-68	FFQ & 24h recall	3911	M	-	18-44	USA	Tertiles	Frequency	Alcohol, Food groups	Carter SJ, Roberts MB, Salter J, Eaton CB. Relationship between Mediterranean Diet Score and atherothrombotic risk: findings from the Third National Health and Nutrition Examination Survey (NHANES III), 1988-1994. Atherosclerosis 2010;210(2):630-6. doi: 10.1016/j.atherosclerosis.2009.12.035.
14	NHANES III	Carter *et al.* , 2010	1988-94	MDS-Attica	0-55	Panagiotakos et al., Nutr Metab Cardiovasc Dis 2006;16:559-68	FFQ & 24h recall	3363	M	-	45-90	USA	Tertiles	Frequency	Alcohol, Food groups	Carter SJ, Roberts MB, Salter J, Eaton CB. Relationship between Mediterranean Diet Score and atherothrombotic risk: findings from the Third National Health and Nutrition Examination Survey (NHANES III), 1988-1994. Atherosclerosis 2010;210(2):630-6. doi: 10.1016/j.atherosclerosis.2009.12.035.
14	NHANES III	Carter *et al.* , 2010	1988-1994	MDS-Attica	0-55	Panagiotakos et al., Nutr Metab Cardiovasc Dis 2006;16:559-68	FFQ & 24h recall	1877	F	-	Post-menopausal	USA	Tertiles	Frequency	Alcohol, Food groups	Carter SJ, Roberts MB, Salter J, Eaton CB. Relationship between Mediterranean Diet Score and atherothrombotic risk: findings from the Third National Health and Nutrition Examination Survey (NHANES III), 1988-1994. Atherosclerosis 2010;210(2):630-6. doi: 10.1016/j.atherosclerosis.2009.12.035.
14	NHANES III	Carter *et al.* , 2010	1988-94	MDS-Attica	0-55	Panagiotakos et al., Nutr Metab Cardiovasc Dis 2006;16:559-68	FFQ & 24h recall	4046	F	-	Pre-menopausal	USA	Tertiles	Frequency	Alcohol, Food groups	Carter SJ, Roberts MB, Salter J, Eaton CB. Relationship between Mediterranean Diet Score and atherothrombotic risk: findings from the Third National Health and Nutrition Examination Survey (NHANES III), 1988-1994. Atherosclerosis 2010;210(2):630-6. doi: 10.1016/j.atherosclerosis.2009.12.035.
15	Guangzhou Nutrition and Health Study (GNHS)	Chen *et al.*, 2016	2008-2010	Alternate Mediterranean Diet Score (aMed)	0-9	Fung *et al.*, AJCN 2005;82:163–73	FFQ	2371	M&F	29.2	59.5 (mean F), 62.1 (mean M), 40-75 (range)	China	Quintiles	Mean	Macronutrients, Food groups	Chen G-d, Dong X-w, Zhu Y-Y, Tian H-y, He J, Chen Y-m. Adherence to the Mediterranean diet is associated with a higher BMD in middle-aged and elderly Chinese. Scientific Reports 2016;6:25662. doi: 10.1038/srep25662
16	ATTICA	Chrysohoou *et al* ., 2004	2001-2002	MDS-Attica	0-55	Ministry of Health and Welfare of Greece, Arch of Hellenic Med 1999;16:516–24	FFQ	1514	M	-	18-87	Attica, Greece	Tertiles	Mean	Alcohol, Food groups	Chrysohoou C, Panagiotakos DB, Pitsavos C, Das UN, Stefanadis C. Adherence to the Mediterranean diet attenuates inflammation and coagulation process in healthy adults: The ATTICA Study. J Am Coll Cardiol 2004;44:152-8.
16	ATTICA	Chrysohoou *et al* ., 2004	2001-2002	MDS-Attica	0-55	Ministry of Health and Welfare of Greece, Arch of Hellenic Med 1999;16:516–24	FFQ	1528	F	-	18-89	Attica, Greece	Tertiles	Mean	Alcohol, Food groups	Chrysohoou C, Panagiotakos DB, Pitsavos C, Das UN, Stefanadis C. Adherence to the Mediterranean diet attenuates inflammation and coagulation process in healthy adults: The ATTICA Study. J Am Coll Cardiol 2004;44:152-8.
17	Swedish Women's Lifestyle and Health cohort (SWLHC)	Couto *et al.*, 2013	1991-1992	MDS [median cut-points]	0-9	Trichopoulou *et al.* BMJ.2005;330:991	FFQ	44840	F	-	30-49	Sweden	Quintiles	Mean	Macronutrients, Food groups	Couto E, Sandin S, Lof M, Ursin G, Adami HO, Weiderpass E. Mediterranean dietary pattern and risk of breast cancer. PLoS One 2013;8(2)
18	Crichton study	Crichton *et al.*, 2013	Not reported	MedDietScore	0-11	Trichopoulou et al. N Engl J Med 2003; 348: 2599–608	FFQ	1183	M&F	36.5	40-65	Australia	Tertiles	Mean	Food groups	Crichton GE, Bryan J, Hodgson JM, Murphy KJ. Mediterranean diet adherence and self-reported psychological functioning in an Australian sample. Appetite 2013:70:53-9
19	Nurses Health Study (NHS)	Crous-Bou *et al.*, 2014	1989-1991	Alternate Mediterranean Diet Score (aMed)	0-9	Fung *et al.*, AJCN 2005;82:163–73	SFFQ	4676	F	-	59 (mean), 42--70 (range)	USA	Quintiles	Mean	Alcohol, Macronutrients, Food groups	Crous-Bou M, Fung TT, Prescott J, Julin B, Du M, Sun Q, Rexrode KM, Hu FB, De Vivo I. Mediterranean diet and telomere length in Nurses' Health Study: population based cohort study. BMJ : British Medical Journal 2014;349
20	Health Professionals follow-up study (HPFS)	de Koning *et al.*, 2011,	1986	Alternate Mediterranean Diet Score (aMed)	0-9	Fung *et al.*, AJCN 2005;82:163–73	FFQ	41615	M	-	Not reported	USA	Quintiles	Mean	Alcohol, Macronutrients	de Koning L, Chiuve SE, Fung TT, Willett WC, Rimm EB, Hu FB. Diet-quality scores and the risk of type 2 diabetes in men. Diabetes Care 2011;34:1150-6.
21	Molise Salute project (MOLI-SA)	di Giuseppe *et al.*, 2008	2005	Mediterranean Adequacy Index	1.2-5.1 (10-90th percentile)	Alberti-Fidanza A, Fidanza F. Public Health Nutr 2004;7:937	SFFQ	282	F	-	18-96	Alto Molise, Italy	tertile	Mean	Alcohol, Food groups	di Giuseppe R, Bonanni A, Olivieri M, Di Castelnuovo A, Donati MB, de Gaetano G, Cerletti C, Iacoviello L. Adherence to Mediterranean diet and anthropometric and metabolic parameters in an observational study in the 'Alto Molise' region: the MOLI-SAL project.
21	Molise Salute project (MOLI-SA)	di Giuseppe *et al.*, 2008	2005	Mediterranean Adequacy Index	1.5-5.4 (10-90th percentile)	Alberti-Fidanza A, Fidanza F. Public Health Nutr 2004;7:937	SFFQ	240	M	-	18-96	Alto Molise, Italy	tertile	Mean	Alcohol, Food groups	di Giuseppe R, Bonanni A, Olivieri M, Di Castelnuovo A, Donati MB, de Gaetano G, Cerletti C, Iacoviello L. Adherence to Mediterranean diet and anthropometric and metabolic parameters in an observational study in the 'Alto Molise' region: the MOLI-SAL project.
22	Seguimiento Universidad de Navarra Follow-up Project (SUN)	Dominguez *et al.*, 2013	1999-2009	MDS [median cut-points]	0-9	Trichopoulou et al. N Engl J Med 2003; 348: 2599–608	FFQ	20155	M&F	39.5	38.4 (mean)	Spain	Quintiles	Frequency	Food groups	Dominguez LJ, Bes-Rastrollo M, de la Fuente-Arrillaga C, Toledo E, Beunza JJ, Barbagallo M, Martinez-Gonzalez MA. Similar prediction of total mortality, diabetes incidence and cardiovascular events using relative- and absolute-component Mediterranean diet score: the SUN cohort. Nutr Metab Cardiovasc Dis 2013;23:451-8.
23	Campanian Postprandial Hyperglycemia Study (CAPRI)	Esposito *et al.*, 2009	2001-2007	MDS [median cut-points]	0-9	Trichopoulou et al. N Engl J Med 2003; 348: 2599–608	SFFQ	901	M&F	51.1	35-70	Campania, Italy	Tertiles	Mean	Alcohol, Food groups	Esposito K, Maiorino MI, Di Palo C, Giugliano D. Adherence to a Mediterranean diet and glycaemic control in Type 2 diabetes mellitus. Diabetic Medicine 2009;26(9):900-7
24	The Three-City study	Feart *et al.*, 2009	1999-2007	MDS [median cut-points]	0-9	Trichopoulou et al. N Engl J Med 2003; 348: 2599–608	FFQ & 24h recall	1410	M&F	37.4	75.9 (mean), 67.6-94.9 (range)	Bordeaux, France	Tertiles	Mean	Food groups	Feart C, Samieri C, Rondeau V, Amieva H, Portet F, Dartigues JF, Scarmeas N, Barberger-Gateau P. Adherence to a Mediterranean diet, cognitive decline, and risk of dementia. Jama 2009;302:638-48.
25	The Three-City study	Feart *et al.*, 2012	2001-2002	MDS [median cut-points]	0-9	Trichopoulou et al. N Engl J Med 2003; 348: 2599–608	FFQ & 24h recall	607	M	-	76.1 (mean)	Bordeaux, France	Tertiles	Mean	Alcohol, Macronutrients, Micronutrients	Feart C, Alles B, Merle B, Samieri C, Barberger-Gateau P. Adherence to a Mediterranean diet and energy, macro-, and micronutrient intakes in older persons. J Physiol Biochem 2012;68:691-700.
25	The Three-City study	Feart *et al.*, 2012	2001-2002	MDS [median cut-points]	0-9	Trichopoulou et al. N Engl J Med 2003; 348: 2599–608	FFQ & 24h recall	988	F	-	76.1 (mean)	Bordeaux, France	Tertiles	Mean	Alcohol, Macronutrients, Micronutrients	Feart C, Alles B, Merle B, Samieri C, Barberger-Gateau P. Adherence to a Mediterranean diet and energy, macro-, and micronutrient intakes in older persons. J Physiol Biochem 2012;68:691-700.
25	The Three-City study	Feart *et al.*, 2012	2001-2002	MDS [median cut-points]	0-9	Trichopoulou et al. N Engl J Med 2003; 348: 2599–608	FFQ & 24h recall	1595	M&F	38.1	76.1 (mean), 67.7-94.9 (range)	Bordeaux, France	Tertiles	Mean	Food groups	Feart C, Alles B, Merle B, Samieri C, Barberger-Gateau P. Adherence to a Mediterranean diet and energy, macro-, and micronutrient intakes in older persons. J Physiol Biochem 2012;68:691-700.
26	Health Professionals follow-up study (HPFS)	Fung *et al* ., 2010	1986	Alternate Mediterranean Diet Score (aMed)	0-9	Fung *et al.*, AJCN 2005;82:163–73	SFFQ	45080	M	-	40–75	USA	Quintiles	Mean	Alcohol, Macronutrients, Micronutrients	Fung TT, Hu FB, Wu K, Chiuve SE, Fuchs CS, Giovannucci E. The Mediterranean and Dietary Approaches to Stop Hypertension (DASH) diets and colorectal cancer. Am J Clin Nutr 2010;92:1429-35.
26	Nurses Health Study (NHS)	Fung *et al.*, 2010	1980	Alternate Mediterranean Diet Score (aMed)	0-9	Fung *et al.*, AJCN 2005;82:163–73	SFFQ	87312	F	-	30-55	USA	Quintiles	Mean	Alcohol, Macronutrients, Micronutrients	Fung TT, Hu FB, Wu K, Chiuve SE, Fuchs CS, Giovannucci E. The Mediterranean and Dietary Approaches to Stop Hypertension (DASH) diets and colorectal cancer. Am J Clin Nutr 2010;92:1429-35.
27	Nurses Health Study (NHS)	Fung *et al.*, 2009	1984-2002	Alternate Mediterranean Diet Score (aMed)	0-9	Fung *et al.*, AJCN 2005;82:163–73	SFFQ	74886	F	-	38-63	USA	Quintiles	Median	Alcohol, Macronutrients, Food groups	Fung TT, Rexrode KM, Mantzoros CS, Manson JE, Willett WC, Hu FB. Mediterranean diet and incidence of and mortality from coronary heart disease and stroke in women. Circulation 2009;119:1093-100.
28	Seguimiento Universidad de Navarra Follow-up Project (SUN)	Galbete *et al.*, 2015	2008	MDS [median cut-points]	0-9	Trichopoulou et al. N Engl J Med 2003; 348: 2599–608	FFQ	823	M&F	71	61.9 (mean)	Spain	Tertiles	Mean	Alcohol, Macronutrients, Food groups	Galbete C, Toledo E, Toledo JB, Bes-Rastrollo M, Buil-Cosiales P, Marti A, Guillen-Grima F, Martinez-Gonzalez MA. Mediterranean diet and cognitive function: the SUN project. J Nutr Health Aging 2015;19:305-12.
29	Health Professionals follow-up study (HPFS)	Gao *et al.*, 2007	1986	Alternate Mediterranean Diet Score (aMed)	0-9	Fung *et al.*, AJCN 2005;82:163–73	SFFQ	49692	M	-	54 (mean)	USA	Quintiles	Mean	Alcohol, Micronutrients,	Gao X, Chen H, Fung TT, Logroscino G, Schwarzschild MA, Hu FB, Ascherio A. Prospective study of dietary pattern and risk of Parkinson disease. Am J Clin Nutr 2007;86:1486-94.
29	Nurses Health Study (NHS)	Gao *et al.*, 2007	1984	Alternate Mediterranean Diet Score (aMed)	0-9	Fung *et al.*, AJCN 2005;82:163–73	SFFQ	81676	F	-	50 (mean)	USA	Quintiles	Mean	Alcohol, Micronutrients,	Gao X, Chen H, Fung TT, Logroscino G, Schwarzschild MA, Hu FB, Ascherio A. Prospective study of dietary pattern and risk of Parkinson disease. Am J Clin Nutr 2007;86:1486-94.
30	Garcia-Meseguer study	Garcia-Meseguer *et al.*, 2014	2012-2013	MDS [median cut-points]	0-10	Trichopoulou et al. N Engl J Med 2003; 348: 2599–608	24h recall	284	M&F	43.7	21.3 (mean)	Castilla-La Mancha, Spain	Tertiles	Mean	Food groups	Garcia-Meseguer MJ, Burriel FC, Garcia CV, Serrano-Urrea R. Adherence to Mediterranean diet in a Spanish university population. Appetite 2014;78:156-64
31	Nurses Health Study (NHS)	Hu *et al.*, 2015	1980–2008	Alternate Mediterranean Diet Score (aMed)	0-9	Fung *et al.*, AJCN 2005;82:163–73	FFQ	83245	F	-	30-55	USA	Quartiles	Frequency	Food groups	Hu Y, Costenbader KH, Gao X, Hu FB, Karlson EW, Lu B. Mediterranean Diet and Incidence of Rheumatoid Arthritis in Women. Arthritis Care & Research 2015;67:597-606.
31	Nurses Health Study II (NHS II)	Hu *et al.*, 2015	1991–2009	Alternate Mediterranean Diet Score (aMed)	0-9	Fung et al., AJCN 2005;82:163–73	SFFQ	91393	F	-	25-42	USA	Quartiles	Frequency	Food groups	Hu Y, Costenbader KH, Gao X, Hu FB, Karlson EW, Lu B. Mediterranean Diet and Incidence of Rheumatoid Arthritis in Women. Arthritis Care & Research 2015;67:597-606.
32	Multiethnic Cohort (MEC)	Jacob *et al.*, 2016	1993-1996	Alternate Mediterranean Diet Score (aMed)	0-9	Fung *et al.* , AJCN 2005;82:163–73	QFFQ	1946	F	-	45-75	Hawaii & California, USA	Quartiles	Median	Alcohol, Food groups	Jacobs S, Harmon BE, Ollberding NJ, Wilkens LR, Monroe KR, Kolonel LN, Le Marchand L, Boushey CJ, Maskarinec G. Among 4 Diet Quality Indexes, Only the Alternate Mediterranean Diet Score Is Associated with Better Colorectal Cancer Survival and Only in African American Women in the Multiethnic Cohort. J Nutr 2016;146(9):1746-55
32	Multiethnic Cohort (MEC)	Jacob *et al.*, 2016	1993-1996	Alternate Mediterranean Diet Score (aMed)	0-9	Fung *et al.* , AJCN 2005;82:163–73	QFFQ	2258	M	-	45-75	Hawaii & California, USA	Quartiles	Median	Alcohol, Food groups	Jacobs S, Harmon BE, Ollberding NJ, Wilkens LR, Monroe KR, Kolonel LN, Le Marchand L, Boushey CJ, Maskarinec G. Among 4 Diet Quality Indexes, Only the Alternate Mediterranean Diet Score Is Associated with Better Colorectal Cancer Survival and Only in African American Women in the Multiethnic Cohort. J Nutr 2016;146(9):1746-55
33	Health Professionals follow-up study (HPFS)	Kenfield *et al.*, 2014	1986	MDS [median cut-points]	0-9	Trichopoulou et al. N Engl J Med 2003; 348: 2599–608	FFQ	4538	M	-	40–75	USA	Tertiles	Mean	Alcohol, Food groups	Kenfield SA, DuPre N, Richman EL, Stampfer MJ, Chan JM, Giovannucci EL. Mediterranean diet and prostate cancer risk and mortality in the Health Professionals Follow-up Study. Eur Urol 2014;65:887-94.
34	Supplementation with Vitamins and Mineral Antioxidants (SU.VI.MAX)	Kesse-Guyot *et al.*, 2013a	1994-2002	Revised Mediterranean Score (MED)	0-11	Issa C *et al.*, . Int J Obes (Lond) 2011 Feb;35(2):251-8	24h recall	3238	M&F	65	49 (mean)	France	Tertiles	Mean	Alcohol, Macronutrients, Micronutrients	Kesse-Guyot E, Ahluwalia N, Lassale C, Hercberg S, Fezeu L, Lairon D. Adherence to Mediterranean diet reduces the risk of metabolic syndrome: A 6-year prospective study. Nutrition, Metabolism and Cardiovascular Diseases 2013;23(7):677-83
35	Supplementation with Vitamins and Mineral Antioxidants (SU.VI.MAX)	Kesse-Guyot *et al.*, 2013b	1994-1996	MDS [median cut-points]	0-9	Trichopoulou et al. N Engl J Med 2003; 348: 2599–608	24h recall	3083	M&F	54	52 (mean)	France	Tertiles	Mean	Macronutrients, Micronutrients,	Kesse-Guyot E, Andreeva VA, Lassale C, Ferry M, Jeandel C, Hercberg S, Galan P. Mediterranean diet and cognitive function: a French study. Am J Clin Nutr 2013;97(2):369-76
35	Supplementation with Vitamins and Mineral Antioxidants (SU.VI.MAX)	Kesse-Guyot *et al.*, 2013b	1994-1996	Mediterranean-Style DietaryPattern Score (MSDPS)	0-100	Rumawas ME et al., J Nutr 2009;139:1150–6	24h recall	3083	M&F	54	52 (mean)	France	Tertiles	Mean	Macronutrients, Micronutrients,	Kesse-Guyot E, Andreeva VA, Lassale C, Ferry M, Jeandel C, Hercberg S, Galan P. Mediterranean diet and cognitive function: a French study. Am J Clin Nutr 2013;97(2):369-76
36	Mammography Cohort (SMC)	Larsson *et al.*, 2017	1997	Modified Mediterranean diet (mMED)	9-45	Trichopoulou et al. N Engl J Med 2003; 348: 2599–608	FFQ	76014	M&F	57	45-79 (range M) & 49-83 (range F)	Sweden	Tertiles	Mean	Food groups	Larsson SC, Hakansson N, Wolk A. Healthy dietary patterns and incidence of biliary tract and gallbladder cancer in a prospective study of women and men. Eur J Cancer 2017;70:42-7
37	Health Professionals follow-up study (HPFS)	Lopez-Garcia *et al.* , 2014	1986	Alternate Mediterranean Diet Score (aMed)	0-9	Fung *et al.* , AJCN 2005;82:163–73	FFQ	6137	M	-	40–75	USA	Quintiles	Mean	Alcohol, Macronutrients, Food groups	Lopez-Garcia E, Rodriguez-Artalejo F, Li TY, Fung TT, Li S, Willett WC, Rimm EB, Hu FB. The Mediterranean-style dietary pattern and mortality among men and women with cardiovascular disease. Am J Clin Nutr 2014;99:172-80.
37	Nurses Health Study (NHS)	Lopez-Garcia *et al.*, 2014	1980	Alternate Mediterranean Diet Score (aMed)	0-9	Fung *et al.* , AJCN 2005;82:163–73	FFQ	11278	F	-	30–55	USA	Quintiles	Mean	Alcohol, Macronutrients, Food groups	Lopez-Garcia E, Rodriguez-Artalejo F, Li TY, Fung TT, Li S, Willett WC, Rimm EB, Hu FB. The Mediterranean-style dietary pattern and mortality among men and women with cardiovascular disease. Am J Clin Nutr 2014;99:172-80
38	Nurses Health Study (NHS)	Mantzoros *et al.*, 2006	1980-1990	MDS [median cut-points]	0-9	Trichopoulou et al. N Engl J Med 2003; 348: 2599–608	FFQ	987	F	-	58.8 (mean)	USA	Tertiles	Mean	Alcohol, Macronutrients, Food groups	Mantzoros CS, Williams CJ, Manson JE, Meigs JB, Hu FB. Adherence to the Mediterranean dietary pattern is positively associated with plasma adiponectin concentrations in diabetic women. Am J Clin Nutr 2006;84:328-35.
39	Seguimiento Universidad de Navarra Follow-up Project (SUN)	Martinez-Gonzalez *et al.*, 2008	1999-	MDS [median cut-points]	0-9	Trichopoulou et al. N Engl J Med 2003; 348: 2599–608	FFQ	13380	M&F	39.7	33.8 (T1 mean), 42.9 (T3 mean)	Spain	Tertiles	Mean	Alcohol, Macronutrients, Food groups	Martinez-Gonzalez MA, de la Fuente-Arrillaga C, Nunez-Cordoba JM, Basterra-Gortari FJ, Beunza JJ, Vazquez Z, Benito S, Tortosa A, Bes-Rastrollo M. Adherence to Mediterranean diet and risk of developing diabetes: prospective cohort study. Bmj 2008;336:1348-51.
40	Seguimiento Universidad de Navarra Follow-up Project (SUN)	Martinez-Gonzalez *et al.*, 2011	1999-2008	MDS [median cut-points]	0-9	Trichopoulou et al. N Engl J Med 2003; 348: 2599–608	FFQ	13609	M&F	40	34 (Q1 mean), 43 (Q5 mean)	Spain	Quintiles	Mean	Alcohol, Macronutrients, Micronutrients, Food groups	Martinez-Gonzalez MA, Garcia-Lopez M, Bes-Rastrollo M, Toledo E, Martinez-Lapiscina EH, Delgado-Rodriguez M, Vazquez Z, Benito S, Beunza JJ. Mediterranean diet and the incidence of cardiovascular disease: a Spanish cohort. Nutr Metab Cardiovasc Dis 2011;21:237-44.
41	EPIC-Spain	Mendez *et al.*, 2006	1992-1996	MDS [median cut-points]	0-8	Trichopoulou et al. N Engl J Med 2003; 348: 2599–608	Diet history	17238	F	-	45.9 (T1 mean), 48.6 (T3 mean)	Spain	Tertiles	Mean	Food groups	Mendez MA, Popkin BM, Jakszyn P, Berenguer A, Tormo MJ, Sanchez MJ, Quiros JR, Pera G, Navarro C, Martinez C, et al. Adherence to a Mediterranean diet is associated with reduced 3-year incidence of obesity. J Nutr 2006;136:2934-8.
41	EPIC-Spain	Mendez *et al.* , 2006	1992-1996	MDS [median cut-points]	0-8	Trichopoulou et al. N Engl J Med 2003; 348: 2599–608	Diet history	10589	M	-	49.1 (T1 mean), 51.8 (T3 mean)	Spain	Tertiles	Mean	Food groups	Mendez MA, Popkin BM, Jakszyn P, Berenguer A, Tormo MJ, Sanchez MJ, Quiros JR, Pera G, Navarro C, Martinez C, et al. Adherence to a Mediterranean diet is associated with reduced 3-year incidence of obesity. J Nutr 2006;136:2934-8.
42	EPIC	Molina-Montes *et al.*, 2017	1990s	Adapted Relative MDS (arMED)	0-16	Trichopoulou et al. N Engl J Med 2003; 348: 2599–608	Country-specific	335060	F	-	51 (mean)	10 European countries	Tertiles	Mean	Alcohol, Macronutrients, Micronutrients, Food groups	Molina-Montes E, Sanchez MJ, Buckland G, Bueno-de-Mesquita HB, Weiderpass E, Amiano P, Wark PA, Kuhn T, Katzke V, Huerta JM, et al. Mediterranean diet and risk of pancreatic cancer in the European Prospective Investigation into Cancer and Nutrition cohort. Br J Cancer 2017;116:811-820.
42	EPIC	Molina-Montes *et al.*, 2017	1990s	Adapted Relative MDS (arMED)	0-16	Trichopoulou et al. N Engl J Med 2003; 348: 2599–608	Country-specific	142249	M	-	52 (mean)	10 European countries	Tertiles	Mean	Alcohol, Macronutrients, Micronutrients, Food groups	Molina-Montes E, Sanchez MJ, Buckland G, Bueno-de-Mesquita HB, Weiderpass E, Amiano P, Wark PA, Kuhn T, Katzke V, Huerta JM, et al. Mediterranean diet and risk of pancreatic cancer in the European Prospective Investigation into Cancer and Nutrition cohort. Br J Cancer 2017;116:811-820.
43	Seguimiento Universidad de Navarra Follow-up Project (SUN)	Nunez-Cordoba *et al.*, 2009	1999-2005	MDS [median cut-points]	0-9	Trichopoulou et al. N Engl J Med 2003; 348: 2599–608	SFFQ	9408	M&F	38.1	34 (mean F), 39.4 (mean M)	Spain	Tertiles	Mean	Alcohol, Macronutrients, Micronutrients, Food groups	Núñez-Córdoba JM, Valencia-Serrano F, Toledo E, Alonso A, Martínez-González MA. The Mediterranean diet and incidence of hypertension: The Seguimiento Universidad de Navarra (SUN) study. American Journal of Epidemiology 2009;169:339-346.
44	ATTICA	Panagiotakos *et al* ., 2006	2001-2002	MDS-Attica	0-55	Ministry of Health and Welfare of Greece, Arch of Hellenic Med 1999;16:516–24	SFFQ	1514	M	-	>18	Attica, Greece	Tertiles	Mean	Alcohol, Macronutrients, Food groups	Panagiotakos DB, Pitsavos C, Stefanadis C. Dietary patterns: a Mediterranean diet score and its relation to clinical and biological markers of cardiovascular disease risk. Nutr Metab Cardiovasc Dis 2006;16:559-68.
44	ATTICA	Panagiotakos *et al* ., 2006	2001-2002	MDS-Attica	0-55	Ministry of Health and Welfare of Greece, Arch of Hellenic Med 1999;16:516–24	SFFQ	1528	F	-	>18	Attica, Greece	Tertiles	Mean	Macronutrients	Panagiotakos DB, Pitsavos C, Stefanadis C. Dietary patterns: a Mediterranean diet score and its relation to clinical and biological markers of cardiovascular disease risk. Nutr Metab Cardiovasc Dis 2006;16:559-68.
45	ATTICA	Pitsavos *et al* ., 2005	2001-2002	MDS-Attica	0-55	Ministry of Health and Welfare of Greece, Arch of Hellenic Med 1999;16:516–24	SFFQ	1517	M	-	45 (mean)	Attica, Greece	Tertiles	Mean	Alcohol, Food groups	Pitsavos C, Panagiotakos DB, Tzima N, Chrysohoou C, Economou M, Zampelas A, Stefanadis C. Adherence to the Mediterranean diet is associated with total antioxidant capacity in healthy adults: the ATTICA study. Am J Clin Nutr 2005;82:694-9.
45	ATTICA	Pitsavos *et al* ., 2005	2001-2002	MDS-Attica	0-55	Ministry of Health and Welfare of Greece, Arch of Hellenic Med 1999;16:516–24	SFFQ	1528	F	-	45 (mean)	Attica, Greece	Tertiles	Mean	Alcohol, Food groups	Pitsavos C, Panagiotakos DB, Tzima N, Chrysohoou C, Economou M, Zampelas A, Stefanadis C. Adherence to the Mediterranean diet is associated with total antioxidant capacity in healthy adults: the ATTICA study. Am J Clin Nutr 2005;82:694-9.
46	China Health and Nutrition Survey (CHNS)	Qin *et al.*, 2015	1989	Adapted MDS	0-9	Trichopoulou et al. N Engl J Med 2003; 348: 2599–608	24h recall	1650	M&F	50	63 (mean)	China	Tertiles	Mean	Alcohol, Food groups	Qin B, Adair LS, Plassman BL, Batis C, Edwards LJ, Popkin BM, Mendez MA. Dietary Patterns and Cognitive Decline Among Chinese Older Adults. Epidemiology 2015;26(5):758-68
47	EPIC-PANACEA	Romaguera *et al.*, 2009	1992-2000	Modified-Mediterranean Diet Score (mMDS)	0-9	Trichopoulou *et al.* BMJ.2005;330:991	Country-specific	145578	M	-	25-70	10 European countries (both regions)	Tertiles	Mean	Alcohol, Macronutrients, Food groups	Romaguera D, Norat T, Mouw T, May AM, Bamia C, Slimani N, Travier N, Besson H, Luan J, Wareham N, et al. Adherence to the Mediterranean diet is associated with lower abdominal adiposity in European men and women. J Nutr 2009;139:1728-37.
47	EPIC-PANACEA	Romaguera *et al.*, 2009	1992-2000	Modified-Mediterranean Diet Score (mMDS)	0-9	Trichopoulou *et al.* BMJ.2005;330:991	Country-specific	351730	F	-	51 (mean)	10 European countries (both regions)	Tertiles	Mean	Alcohol, Macronutrients, Food groups	Romaguera D, Norat T, Mouw T, May AM, Bamia C, Slimani N, Travier N, Besson H, Luan J, Wareham N, et al. Adherence to the Mediterranean diet is associated with lower abdominal adiposity in European men and women. J Nutr 2009;139:1728-37.
48	Italian Network of case-control studies	Rossi *et al.* 2008	1991-2001	MDS [median cut-points]	0-8	Trichopoulou et al. N Engl J Med 2003; 348: 2599–608	FFQ	3090	M	-	58 (median)	Italy	Quintiles	Mean	Alcohol, Food groups	Rossi M, Negri E, Bosetti C, Dal Maso L, Talamini R, Giacosa A, Montella M, Franceschi S, La Vecchia C. Mediterranean diet in relation to body mass index and waist-to-hip ratio. Public Health Nutr 2008;11(2):214-7
48	Italian Network of case-control studies	Rossi *et al.* 2008	1991-2001	MDS [median cut-points]	0-8	Trichopoulou et al. N Engl J Med 2003; 348: 2599–608	FFQ	3529	F	-	59 (median)	Italy	Quintiles	Mean	Alcohol, Food groups	Rossi M, Negri E, Bosetti C, Dal Maso L, Talamini R, Giacosa A, Montella M, Franceschi S, La Vecchia C. Mediterranean diet in relation to body mass index and waist-to-hip ratio. Public Health Nutr 2008;11(2):214-7
49	EPIC-Greece	Rossi *et al.*, 2013	1994-1999	MDS [median cut-points]	0-9	Trichopoulou et al. N Engl J Med 2003; 348: 2599–608	SFFQ	22295	M&F	40	50 (median)	Greece	Quartiles	Median	Macronutrients	Rossi M, Turati F, Lagiou P, Trichopoulos D, Augustin LS, La Vecchia C, Trichopoulou A. Mediterranean diet and glycaemic load in relation to incidence of type 2 diabetes: results from the Greek cohort of the population-based European Prospective Investigation into Cancer and Nutrition (EPIC). Diabetologia 2013;56:2405-13.
50	Framingham Offspring Cohort	Rumawas *et al.*, 2009	1998-2001	Mediterranean-Style Dietary Pattern Score (MSDPS)	0-100	Ministry of Health and Welfare of Greece, Arch of Hellenic Med 1999;16:516–24	SFFQ	3021	M&F	Q1=55, Q5=35	60.2 (Q1 mean), 61.5 (Q5 mean)	USA	Quintiles	Mean	Alcohol, Macronutrients, Micronutrients	Rumawas ME, Dwyer JT, McKeown NM, Meigs JB, Rogers G, Jacques PF. The development of the Mediterranean-style dietary pattern score and its application to the American diet in the Framingham Offspring Cohort. J Nutr 2009;139(6):1150-6
51	Women's Health Study (WHS)	Samieri *et al.*, 2013a	1998	Alternate Mediterranean Diet Score (aMed)	0-9	Fung TT *et al* ., Circulation 2009;119(8):1093-1100	SFFQ	6174	F	-	66 (mean)	USA	Quintiles	Mean	Alcohol, Food groups	Samieri C, Grodstein F, Rosner BA, Kang JH, Cook NR, Manson JE, Buring JE, Willett WC, Okereke OI. Mediterranean diet and cognitive function in older age. Epidemiology 2013;24(4):490-9
52	Nurses Health Study (NHS)	Samieri *et al.*, 2013	1984-1986	Alternate Mediterranean Diet Score (aMed)	0-9	Fung *et al.* , AJCN 2005;82:163–73	SFFQ	16058	F	-	74 (mean)	USA	Quintiles	Mean	Alcohol, Food groups	Samieri C, Okereke OI, E ED, Grodstein F. Long-term adherence to the Mediterranean diet is associated with overall cognitive status, but not cognitive decline, in women. J Nutr 2013;143:493-9.
53	Seguimiento Universidad de Navarra Follow-up Project (SUN)	Sanchez-Villegas *et al.*, 2006	1999	Mediterranean dietary pattern (MDP)	10-30	Trichopoulou et al. N Engl J Med 2003; 348: 2599–608	SFFQ	6319	M&F	42.4	37 (mean)	Spain	Quartiles	Mean	Alcohol, Macronutrients, Micronutrients	Sanchez-Villegas A, Bes-Rastrollo M, Martinez-Gonzalez MA, Serra-Majem L. Adherence to a Mediterranean dietary pattern and weight gain in a follow-up study: the SUN cohort. Int J Obes (Lond) 2006;30:350-8.
54	Seguimiento Universidad de Navarra Follow-up Project (SUN)	Sanchez-Villegas *et al.*, 2009	1999	MDS [median cut-points]	0-9	Trichopoulou et al. N Engl J Med 2003; 348: 2599–608	SFFQ	10094	M&F	41.6	37 (mean)	Spain	Quintiles	Mean	Alcohol, Macronutrients, Food groups	Sanchez-Villegas A, Delgado-Rodriguez M, Alonso A, Schlatter J, Lahortiga F, Serra Majem L, Martinez-Gonzalez MA. Association of the Mediterranean dietary pattern with the incidence of depression: the Seguimiento Universidad de Navarra/University of Navarra follow-up (SUN) cohort. Arch Gen Psychiatry 2009;66:1090-8.
55	Washington Heights-Inwood Columbia Aging Project (WHICAP)	Scarmeas *et al.*, 2006	1992 & 1999	MDS [median cut-points]	0-9	Trichopoulou et al. N Engl J Med 2003; 348: 2599–608	SFFQ	2258	M&F	33	77 (mean)	New York, USA	Tertiles	Median	Food groups	Scarmeas N, Stern Y, Tang MX, Mayeux R, Luchsinger JA. Mediterranean diet and risk for Alzheimer's disease. Ann Neurol 2006;59(6):912-21.
56	REgistre Gironí del COR (Girona Heart Registry) (REGICOR)	Schroder *et al.*, 2004	1999-2000	MDS [tertile cut-points]	9-27	Trichopoulou et al. N Engl J Med 2003; 348: 2599–608	FFQ	1615	F	-	50.1 (mean) 25-74 (range)	Girona, Spain	Quartiles	Mean	Macronutrients, Food groups	Schroder H, Marrugat J, Vila J, Covas MI, Elosua R. Adherence to the traditional mediterranean diet is inversely associated with body mass index and obesity in a spanish population. J Nutr 2004;134(12):3355-61
56	REgistre Gironí del COR (Girona Heart Registry) (REGICOR)	Schroder *et al.*, 2004	1999-2000	MDS [tertile cut-points]	9-27	Trichopoulou et al. N Engl J Med 2003; 348: 2599–608	FFQ	1547	M	-	50.4 (mean) 25-74 (range)	Girona, Spain	Quartiles	Mean	Macronutrients, Food groups	Schroder H, Marrugat J, Vila J, Covas MI, Elosua R. Adherence to the traditional mediterranean diet is inversely associated with body mass index and obesity in a spanish population. J Nutr 2004;134(12):3355-61
57	Health, Aging, and Body Composition cohort (Health-ABC)	Shahar *et al.*, 2012,	1998-1999	MDS [median cut-points]	0-9	Trichopoulou et al. N Engl J Med 2003; 348: 2599–608	FFQ	2225	M&F	52	74.5 (mean), 70-79 (range)	USA	Tertiles	Mean	Alcohol, Food groups	Shahar DR, Houston DK, Hue TF, Lee JS, Sahyoun NR, Tylavsky FA, Geva D, Vardi H, Harris TB. Adherence to mediterranean diet and decline in walking speed over 8 years in community-dwelling older adults. J Am Geriatr Soc 2012;60(10):1881-8
58	InCHIANTI	Talegawkar *et al.*, 2012,	1998-1999	MDS [median cut-points]	0-9	Trichopoulou et al. N Engl J Med 2003; 348: 2599–608	FFQ	690	M&F	48.3	73 (mean)	Tuscany, Italy	Tertiles	Mean	Alcohol, Macronutrients, Micronutrients, Food groups	Talegawkar SA, Bandinelli S, Bandeen-Roche K, Chen P, Milaneschi Y, Tanaka T, Semba RD, Guralnik JM, Ferrucci L. A Higher Adherence to a Mediterranean-Style Diet Is Inversely Associated with the Development of Frailty in Community-Dwelling Elderly Men and Women. The Journal of Nutrition 2012;142:2161-2166
59	Swedish Mammography Cohort (SWC)	Tektonidis *et al.*, 2015	1997	Modified Mediterranean diet (mMED)	0-8	Trichopoulou et al. N Engl J Med 2003; 348: 2599–608	FFQ	32921	F	-	48-83	Sweden	Quartiles	Mean	Alcohol, Food groups	Tektonidis TG, Akesson A, Gigante B, Wolk A, Larsson SC. A Mediterranean diet and risk of myocardial infarction, heart failure and stroke: A population-based cohort study. Atherosclerosis 2015;243:93-8.
60	Cohort of Swedish Men (CoSM)	Tektonidis *et al* ., 2016	1997	Modified Mediterranean diet (mMED)	0-8	Trichopoulou et al. N Engl J Med 2003; 348: 2599–608	FFQ	37308	M	-	59 (mean)	Sweden	Quartiles	Mean	Alcohol, Food groups	Tektonidis TG, Akesson A, Gigante B, Wolk A, Larsson SC. Adherence to a Mediterranean diet is associated with reduced risk of heart failure in men. Eur J Heart Fail 2016;18:253-9.
61	Nurses Health Study II (NHS II)	Tobias *et al.*, 2012	1991	Alternate Mediterranean Diet Score (aMed)	0-8	Fung *et al.* , AJCN 2005;82:163–73	SFFQ	4413	F	-	37.5 (mean)	USA	Quartiles	Mean	Alcohol, Macronutrients	Tobias DK, Hu FB, Chavarro J, Rosner B, Mozaffarian D, Zhang C. Healthful dietary patterns and type 2 diabetes mellitus risk among women with a history of gestational diabetes mellitus. Archives of Internal Medicine 2012;172:1566-1572.
62	EPIC-Greece	Trichopoulou *et al.*, 2005	1994-1999	MDS [median cut-points]	0-9	Trichopoulou et al. N Engl J Med 2003; 348: 2599–608	SFFQ	9612	M	-	20-86	Greece	Quartiles	Mean	Alcohol, Macronutrients, Food groups	Trichopoulou A, Naska A, Orfanos P, Trichopoulos D. Mediterranean diet in relation to body mass index and waist-to-hip ratio: the Greek European Prospective Investigation into Cancer and Nutrition Study. Am J Clin Nutr 2005;82:935-40.
62	EPIC-Greece	Trichopoulou *et al.*, 2005	1994-1999	MDS [median cut-points]	0-9	Trichopoulou et al. N Engl J Med 2003; 348: 2599–608	SFFQ	13985	F	-	20-87	Greece	Quartiles	Mean	Alcohol, Macronutrients, Food groups	Trichopoulou A, Naska A, Orfanos P, Trichopoulos D. Mediterranean diet in relation to body mass index and waist-to-hip ratio: the Greek European Prospective Investigation into Cancer and Nutrition Study. Am J Clin Nutr 2005;82:935-40.
63	Minnesota Breast Cancer Family Study (MBCFS)	Tseng *et al.*, 2008	1990	MDS [median cut-points]	0-9	Trichopoulou et al. N Engl J Med 2003; 348: 2599–608	SFFQ	1286	F	-	57 (mean)	Minnesota, USA	Tertiles	Mean	Alcohol, Food groups	Tseng M, Sellers TA, Vierkant RA, Kushi LH, Vachon CM. Mediterranean diet and breast density in the Minnesota Breast Cancer Family Study. Nutr Cancer 2008;60(6):703-9
64	Women's Health Initiative (WHI)	Vargas *et al.*, 2016	1993	Alternate Mediterranean Diet Score (aMed)	0-9	Fung TT *et al* ., Circulation 2009;119(8):1093-1100	FFQ	78273	F	-	63 (mean)	USA	Quintiles	Mean	Alcohol, Macronutrients, Food groups	Vargas AJ, Neuhouser ML, George SM, Thomson CA, Ho GY, Rohan TE, Kato I, Nassir R, Hou L, Manson JE. Diet Quality and Colorectal Cancer Risk in the Women's Health Initiative Observational Study. Am J Epidemiol 2016;184(1):23-32
65	Cache County Study on Memory, Health and Aging (CCMS)	Wengreen *et al.*, 2013	1995-1996	Mediterranean Diet Accordance score	0-9	Fung *et al.*, AJCN 2005;82:163–73	FFQ	3831	M&F	42.9	74 (mean)	Utah, USA	Quintiles	Mean	Macronutrients, Micronutrients,	Wengreen H, Munger RG, Cutler A, Quach A, Bowles A, Corcoran C, Tschanz JT, Norton MC, Welsh-Bohmer KA. Prospective study of Dietary Approaches to Stop Hypertension- and Mediterranean-style dietary patterns and age-related cognitive change: the Cache County Study on Memory, Health and Aging. Am J Clin Nutr 2013;98(5):1263-71.
66	REasons for Geographic and Racial Differences in Stroke (REGARDS)	Whalen *et al.*, 2017	2003-2007	Mediterranean diet pattern score	11-55	-	FFQ	21423	M&F	44	65 (mean)	USA	Quintiles	Mean	Alcohol, Macronutrients	Whalen KA, Judd S, McCullough ML, Flanders WD, Hartman TJ, Bostick RM. Paleolithic and Mediterranean Diet Pattern Scores Are Inversely Associated with All-Cause and Cause-Specific Mortality in Adults. J Nutr 2017;147(4):612-20.
